# Highly Robust Membrane Electrode Assembly Using Engineered Dual‐Phase Iridium Oxide Catalysts Breaking the Activity–Durability Trade‐Off in Proton Exchange Membrane Water Electrolysis

**DOI:** 10.1002/advs.202524243

**Published:** 2026-03-10

**Authors:** Ho Seong Yang, Song Gyun Kim, Rubin Shin, Youngin Cho, Chanho Pak

**Affiliations:** ^1^ Graduate School of Energy Convergence Gwangju Institute of Science and Technology Gwangju Republic of Korea; ^2^ Department of Chemistry Gwangju Institute of Science and Technology Gwangju Republic of Korea

**Keywords:** activity‐durability trade‐off, dual phase catalyst, iridium oxide catalyst, oxygen evolution reaction, proton exchange membrane water electrolysis

## Abstract

Proton exchange membrane water electrolysis (PEMWE) is a compelling route for sustainable green hydrogen production. Yet Ir‐based oxygen evolution reaction (OER) catalysts suffer from a persistent trade‐off between activity and stability under acidic conditions. Amorphous IrO_x_ displays high intrinsic activity but limited structural resilience, whereas crystalline IrO_2_ affords robustness at the expense of accessible active sites. Reconciling these conflicting properties through rational structural design remains a significant challenge. This study presents a hollow dual‐phase iridium oxide (HDP‐IrO_x_) catalyst comprising coexisting amorphous and crystalline domains, synthesized via polydopamine‐coated polystyrene‐sphere hard templates. Calcination temperature precisely tunes the amorphous–crystalline ratios, while template diameter (190, 240, and 360 nm) controls shell architecture and the extent of amorphous domain formation. The optimized HDP‐IrO_x_‐240 exhibits a low overpotential of 283 mV at 10 mA cm^−2^ in half‐cell evaluation, and HDP‐IrO_x_‐360 achieves 1.77 V at 2 A cm^−2^ in single‐cell operation with remarkable stability (31.5 µV h^−1^ decay rate) over 1036 h. Cooperative amorphous–crystalline interactions ensure efficient charge transport while maintaining structural integrity. Dual‐phase engineering thereby establishes an intrinsic design paradigm that reconciles activity and durability. This approach advances next‐generation PEMWE anodes with low Ir loading.

## Graphical abstract

The dual‐phase iridium oxide catalyst was synthesized via a hard‐template‐assisted method, overcoming the conventional activity–durability trade‐off observed in Ir‐based catalysts. The HDP‐IrO_x_ exhibits excellent catalytic activity and long‐term stability through the synergistic coupling between amorphous and crystalline phases, offering new insights into the design of Ir‐based catalysts for PEMWE.

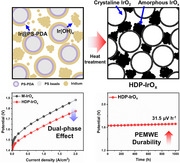

## 1 Introduction

The importance of achieving carbon neutrality and using renewable energy in response to accelerating climate change has been increasingly emphasized. Recently, the deployment of renewable energy facilities such as solar, wind, and hydroelectric power has been steadily increasing [1], but such renewable energy is constrained by the intermittency of electricity generation. Therefore, technology that can efficiently store electrical energy to address load fluctuations is essential [2]. Recently, a method of producing green hydrogen that uses electricity generated from renewable energy to electrolyze water and store the hydrogen has attracted considerable interest [3, 4, 5]. This method offers a significant advantage in effectively converting surplus electricity into hydrogen, enabling long‐term storage. Currently, various water electrolysis technologies have been extensively studied, among which proton exchange membrane water electrolysis (PEMWE) is the most intensively investigated technology due to its advantages, including high current density and efficiency, high‐purity hydrogen production, and fast response time [6, 7].

There are various core materials in PEMWE systems, but a key material that determines their performance is the anode catalyst, which directly contributes to the oxygen evolution reaction (OER) [8, 9, 10]. Currently, iridium (Ir)‐based catalysts are primarily employed at the anode, and their development has been extensively investigated. However, Ir is a precious metal with scarce and costly reserves, and efforts to reduce its usage have been actively pursued via research and development [11]. The goal of the study is to simultaneously ensure durability and activity while reducing Ir amounts in the anode [12]. The catalytic activity of Ir is primarily derived from amorphous iridium oxide. Amorphous IrO_x_ contains uneven Ir─O bonds and abundant oxygen vacancies [13, 14]. It also contains abundant Ir^3+^/Ir^4+^ redox species, leading to the formation of unsaturated Ir coordination sites [15, 16]. Such unsaturated Ir sites can readily participate in electron/proton transfer and serve as active sites. These disordered Ir─O bonds and abundant oxygen vacancies promote the lattice oxygen mechanism (LOM), in which lattice oxygen directly participates during the OER, rather than the adsorbate evolution mechanism (AEM), where the OER proceeds via adsorbed oxygen intermediates. In LOM, lattice oxygen contributes to the formation of O─O bonds to decrease the reaction barrier and enhance catalytic activity, but at the same time, they lead to the formation of oxygen vacancies and structural collapse of the Ir–O network, thereby accelerating Ir dissolution [17, 18]. Therefore, amorphous IrO_x_ exhibits poor durability owing to its reduced structural stability despite high initial activity. In contrast, crystalline IrO_2_ possesses strong Ir─O bonds and remains structurally stable [19]. This suggests that the catalytic activity is limited by the low density of unsaturated Ir sites and oxygen vacancies. Crystalline IrO_2_ primarily follows the AEM for OER. In this mechanism, oxygen is generated via the sequential formation and conversion of adsorbed intermediates (OH^*^, O^*^, and OOH^*^), with lattice oxygen not directly participating in the reaction [18, 20]. Therefore, although the reaction barrier is relatively high and the initial activity is low, the Ir–O network is maintained, and Ir dissolution is suppressed. As a result, crystalline IrO_2_ consequently exhibits high stability [21].

Therefore, combining the intrinsic activity of amorphous IrO_x_ with the superior stability of crystalline IrO_2_ has emerged as a central challenge in the design of Ir‐based OER catalysts. Such an approach enables the development of next‐generation catalysts that can simultaneously secure high activity and long‐term durability under limited Ir resources, thereby enhancing the cost‐effectiveness of PEMWE systems [22]. However, the simultaneous integration of two contrasting properties within a single catalyst structure presents inherently conflicting material challenges. Therefore, to simultaneously achieve the high activity of amorphous IrO_x_ and the stability of crystalline IrO_2_, sophisticated design strategies are required, such as introducing amorphous‐crystalline interfaces [23, 24, 25] or spatially integrating different phases [26, 27]. Such structures can provide long‐term structural stability by offering unsaturated Ir sites and oxygen vacancies favorable for the OER at the surface, while maintaining a stable Ir‐O network internally [28]. These several constraints explain why catalysts simultaneously exhibiting high activity and durability are limited to elaborate structural designs, such as support‐based binary catalysts [29, 30, 31, 32], partially crystallized IrO_x_ [33, 34, 35, 36], or heteroatom‐doped IrO_x_ [37, 38].

In this study, a hollow dual‐phase iridium oxide (HDP‐IrO_x_) catalyst, comprising both crystalline and amorphous phases, was synthesized via a template‐assisted method. This study offers a novel design strategy to mitigate the trade‐off between activity and stability by simultaneously incorporating dual‐phase and hollow structures in the IrO_x_ catalyst. Such 3D hollow nanostructures can maximize the number of active sites by providing a high specific surface area, continuous charge transport, and mass transfer pathways, while concurrently improving gas bubble detachment and structural integrity [39, 40]. The structure consists of crystalline IrO_2_ with a hollow morphology, with smaller amorphous IrO_x_ spheres located between the crystalline hollow spheres. The hollow structure facilitates water transport and the escape of oxygen bubbles, thereby sustaining the exposure of active sites [41]. Simultaneously, the crystalline hollow spheres stably support Ir─O bonds, ensuring continuous conduction pathways and structural durability, while the amorphous IrO_x_ domains provide abundant active sites. The catalyst produced in this method achieved a cell voltage of 1.77 V at 2 A cm^−2^ with an Ir loading of 0.2 mg_Ir_ cm^−2^, exhibiting a low degradation rate (≈ 31.5 µV h^−1^) over 1000 h. This study represents the first synthesis approach for Ir‐based catalysts featuring coexisting crystalline and amorphous phases. It provides an innovative design paradigm that introduces a hollow structure to maximize Ir utilization efficiency while overcoming the trade‐off between activity and durability.

## 2 Results and Discussion

### 2.1 Synthesis and Morphological Overview of Dual‐Phase Hollow Iridium Oxide

**FIGURE 1:**
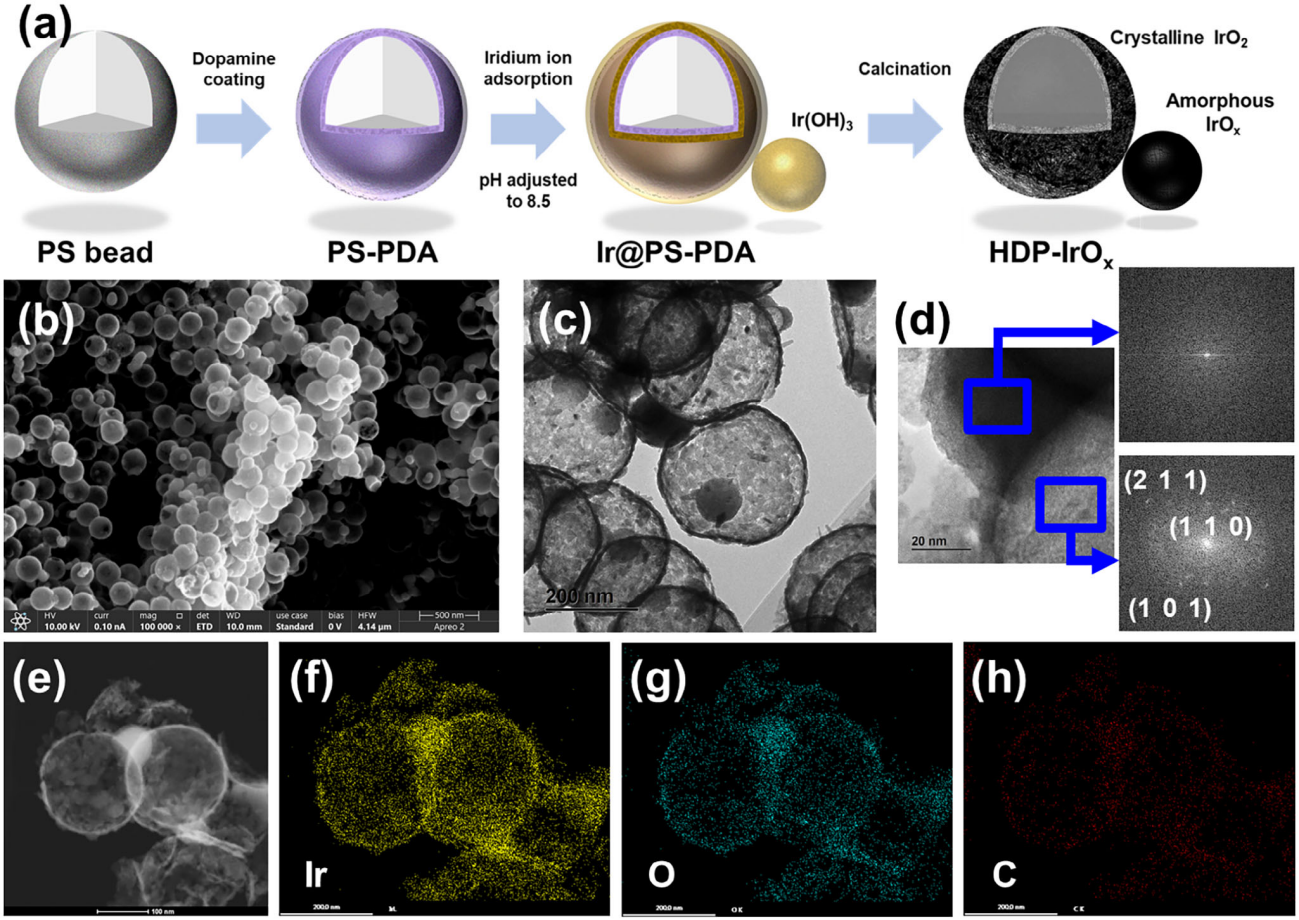
(a) Schematic illustration of the synthesis process for the HDP‐IrO_x_ catalyst, (b) scanning electron microscoy (SEM) image of HDP‐IrO_x_, (c,d) Transmission electron microscopy (TEM) images of HDP‐IrO_x_ with low and high resolution images with corresponding FFT patterns, (e,f,g,h) scanning transmission electron microscopy (STEM) image and energy dispersive X‐ray spectroscopy mapping of HDP‐IrO_x._

Hollow dual‐phase iridium oxide (HDP‐IrO_x_) was synthesized using polydopamine (PDA)‐coated polystyrene (PS) beads as a template (Figure 1a; Figure S1). In previous studies, metal oxides have been synthesized using PS beads as hard templates [30, 39]. The size of the PS beads can also be tuned by varying the amount of sodium dodecyl sulfate (SDS), allowing the preparation of hard templates with different sizes (Figure S2). In this study, PDA was coated on the surface to immobilize metal ions via the catechol groups, dopamine's functional moieties [42]. Under basic conditions, the hydroxyl groups of catechol can be ionized to chelate metal cations. The PDA coating on the surface of PS beads was confirmed by FT‐IR analysis (Figure S3). The aromatic ring vibration of polystyrene was observed at 1454 cm^−1^. Additional peaks were identified at 1030 cm^−1^ corresponding to C‐O stretching, at 1125 cm^−1^ attributed to amine groups of dopamine [43], and at 1295 cm^−1^ associated with the PDA structure [44]. In addition, as shown in Figure S4, the zeta potential of PS‐PDA was higher than that of pristine PS beads over the entire pH range. This is because the anionic surfactant SDS imparts a strong negative charge to the PS surface, whereas the subsequently coated PDA layer partially shields and neutralizes these charges. The amine and hydroxyl groups of PDA modify the surface charge distribution, resulting in a decrease in the overall negative potential. Such a shift in zeta potential is consistent with the FT‐IR results and further supports the successful coating of PDA on the PS bead surface. TEM analysis was conducted to assess surface morphology, but no apparent change was observed (Figure S5).

To immobilize iridium cations on catechol groups, a buffer solution was used to maintain the pH range of 8.5–9.5. After the immobilization of iridium on catechol groups, the remaining iridium ions could precipitate as Ir(OH)_3_. This reaction occurs during the reflux process. The sample was then calcined in an air atmosphere, thereby removing the template. Simultaneously, the immobilized Ir was converted into hollow iridium oxide, while the precipitated Ir(OH)_3_ was transformed into amorphous iridium oxide (Figure 1b,c). FFT analysis of TEM images confirmed that the smaller dark spheres were amorphous, while the larger hollow spheres exhibited crystallinity (Figure 1d). This confirms the successful synthesis of a dual‐phase catalyst, in which amorphous IrO_x_ domains are located between the crystalline hollow IrO_2_ spheres. This phase distinction was further validated not merely by indirect FFT‐based analysis, but by atomic‐resolution Cs‐corrected (S)TEM observations, which enabled direct visualization of lattice fringes in the crystalline IrO_2_ domains, while simultaneously confirming the absence of lattice fringes in the amorphous IrO_x_ regions in real space. In particular, the interfacial regions between the crystalline hollow IrO_2_ spheres and the amorphous IrO_x_ domains located in between were clearly observed, providing direct microstructural evidence that the catalyst possesses a dual‐phase architecture in which amorphous and crystalline phases spatially coexist (Figure S6). Energy‐dispersive X‐ray spectroscopy (EDS) analysis revealed characteristic peaks of Ir in the small spheres, confirming the presence of IrO_x_ (Figure 1e–h). When PS beads coated with PDA was used for synthesis, HDP‐IrO_x_‐*n* with a hollow dual‐phase structure was formed. In contrast, M‐IrO_x_‐*n* with a microporous amorphous structure was obtained from PS beads without PDA coating. In this notation, *n* (*n* = 190, 240, or 360 nm) represents the diameter of the PS template used. In the absence of PDA, iridium cations could not be immobilized, and Ir(OH)_3_ precipitated around the PS beads in a basic environment (Figure S7). At low iridium precursor concentrations, the template surface was not fully covered, leading to the coexistence of a skin‐like morphology without hollow spheres and amorphous iridium oxide (Figure S8).

Nitrogen (N_2_) adsorption–desorption isotherms were measured to evaluate changes in specific surface area (SSA) before and after heat treatment (Figure S9). Before heat treatment, no distinct hysteresis was observed, whereas after heat treatment, an H1‐type loop emerged. This indicates the formation of relatively uniform spherical particles and suggests improvements in pore‐size uniformity and interconnectivity [45]. These results indicate that the template was removed during calcination, resulting in the formation of internal pores while maintaining the hollow structure. Regarding the SSA, the internal pores were not directly connected to the exterior, showing no significant difference, though there was a slight increase. Based on N_2_ adsorption–desorption analysis, it was confirmed that the hollow structure was successfully formed by template removal during heat treatment. Subsequently, analyses at different heat treatment temperatures were conducted to investigate the role and contribution of amorphous iridium oxide in the dual‐phase catalyst.

### 2.2 Impact of Calcination Temperature on Phase and Surface Chemistry

**FIGURE 2:**
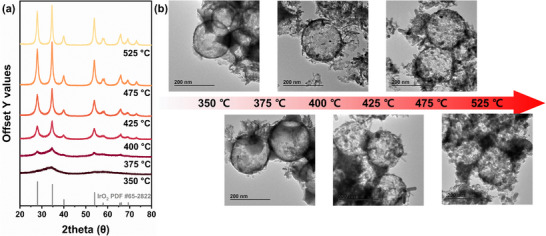
Structural characterization of HDP‐IrO_x_ catalysts as a function of calcination temperature: (a) X‐ray diffraction patterns, and (b) TEM images of catalysts calcined at different temperatures.

The effect of heat‐treatment (calcination) temperature on the formation of a dual‐phase hybrid structure and its correlation with electrochemical activity was clarified. Calcination was conducted at 350°C–525°C because the template could not be completely removed below 350°C. X‐ray diffraction (XRD) analysis (Figure 2a) revealed that increasing the calcination temperature enhanced crystallinity, consistent with TEM observations. Specifically, at 350°C, the hollow spheres remained amorphous, whereas crystallinity began on the hollow sphere surfaces at 375°C, while amorphous IrO_x_ persisted in the smaller filled spheres. This dual‐phase structure, where crystalline and amorphous phases coexist, could be attributed to the crystallization temperature difference between dopamine‐bound iridium and amorphous IrO_x_ derived from Ir(OH)_3_. XRD and TEM further confirmed that all regions were converted to crystalline structures at temperatures above 400°C (Figure 2b).

**FIGURE 3:**
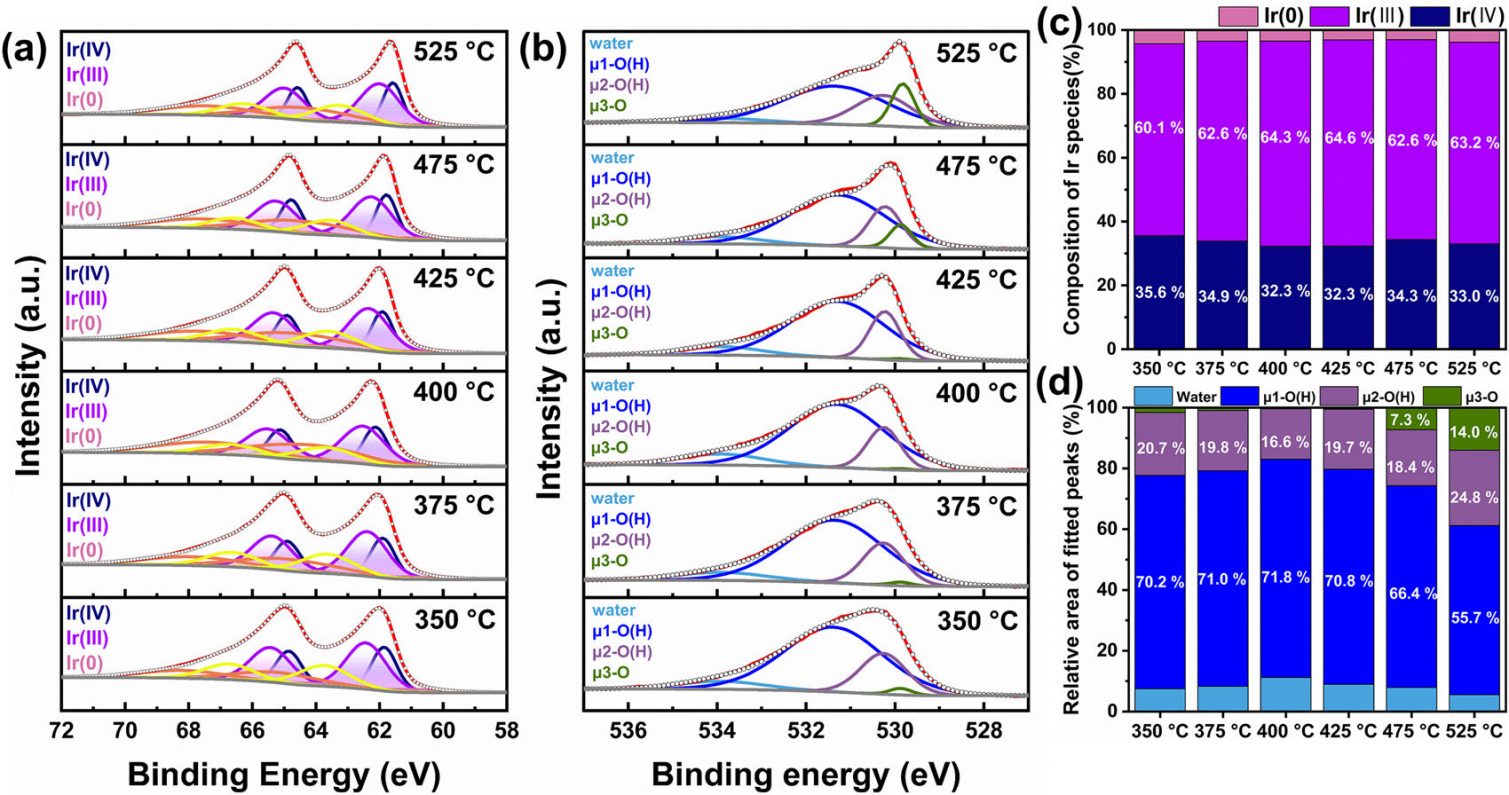
Chemical characterization of HDP‐IrO_x_ catalysts as a function of calcination temperature: (a) Ir 4f X‐ray photoelectron spectroscopy spectra, (b) O 1s spectra, (c) composition of ratio of Ir oxidation states, and (d) relative area of O bonding species.

The effect of heat treatment temperature on the oxidation state of iridium was subsequently investigated by XPS (Figure 3a,c). In general, the stability and activity of IrO_x_ catalysts strongly depend on the oxidation state of Ir, and a trade‐off exists between the two properties. In all heat treatment conditions, the Ir^3+^ ratio was maintained at 60% or higher, indicating its dominance over Ir^4+^, which is relatively unfavorable for activity. Previous studies have reported that Ir^3+^ has a strong binding energy to intermediates during OER and thereby contributes to higher activity [46]. Notably, the Ir^3+^ ratio remained nearly unchanged with increasing calcination temperature, confirming that the synthesized catalyst maintains a stable surface oxidation state under these conditions.

In addition, the O 1s spectrum was deconvoluted to identify oxygen species on the catalyst surface (Figure 3b,d). The species were classified into μ_1_, μ_2_, and μ_3_ based on the Ir─O bonding coordination [47], where μ_1_ corresponds to oxygen singly bonded to metal ions, μ_2_ to oxygen in double‐bonded states, and μ_3_ to lattice oxygen (Figure S10). The corresponding binding energies were identified as μ_1_ (531.27–531.39 eV), μ_2_ (530.22–530.26 eV), and μ_3_ (529.81–529.87 eV). The μ_1_ peak was predominant in amorphous IrO_x_, whereas the μ_2_ and μ_3_ peaks were predominant in crystalline IrO_2_. With increasing heat‐treatment temperature, the μ_1_ peak intensity decreased and the μ_3_ peak intensity increased, reflecting the gradual transformation of amorphous IrO_x_ into crystalline IrO_2_. While TEM primarily reveals the bulk crystal structure, XPS is interpreted as reflecting amorphous characteristics due to its sensitivity to the thin surface defect layer. Hence, these results indicate that the structural evolution of IrO_x_ can be more comprehensively understood through the complementary both techniques.

Meanwhile, although XRD and TEM analyses clearly demonstrate an enhancement in the crystallinity of IrO_2_ with increasing calcination temperature, the surface Ir^3+^ fraction derived from XPS is maintained or slightly increased. This observation is not contradictory when considering that crystallinity enhancement and surface chemical evolution can occur simultaneously at different length scales. As XPS is a surface‐sensitive technique probing only the top few nanometers of the catalyst surface, the oxidation‐state distribution obtained from XPS primarily reflects localized chemical environments at the surface. Indeed, the O 1s XPS spectra show that with increasing calcination temperature, the relative contribution of lattice oxygen decreases, whereas the fraction of defect‐related and hydroxyl‐associated oxygen species increases. This trend suggests that surface dehydration and oxygen rearrangement during high‐temperature calcination lead to the formation of surface oxygen vacancies, which require local charge compensation and thereby induce partial reduction of Ir^4+^ to Ir^3+^ in the near‐surface region. Consequently, these results indicate the formation of a structure in which a crystalline IrO_2_ coexists with a defect‐rich, Ir^3+^‐enriched surface.

N_2_ adsorption–desorption isotherms were further measured to evaluate the hollow structure of the catalyst and estimate its SSA. TEM observations revealed that with increasing calcination temperature, Ir growth occurred, suggesting a corresponding increase in SSA. However, the difference in SSA was negligible, likely due to crystallization and aggregation of amorphous iridium oxide. The nitrogen adsorption isotherms confirmed that all samples exhibited the hollow structure observed in TEM (Figure S11).

**FIGURE 4:**
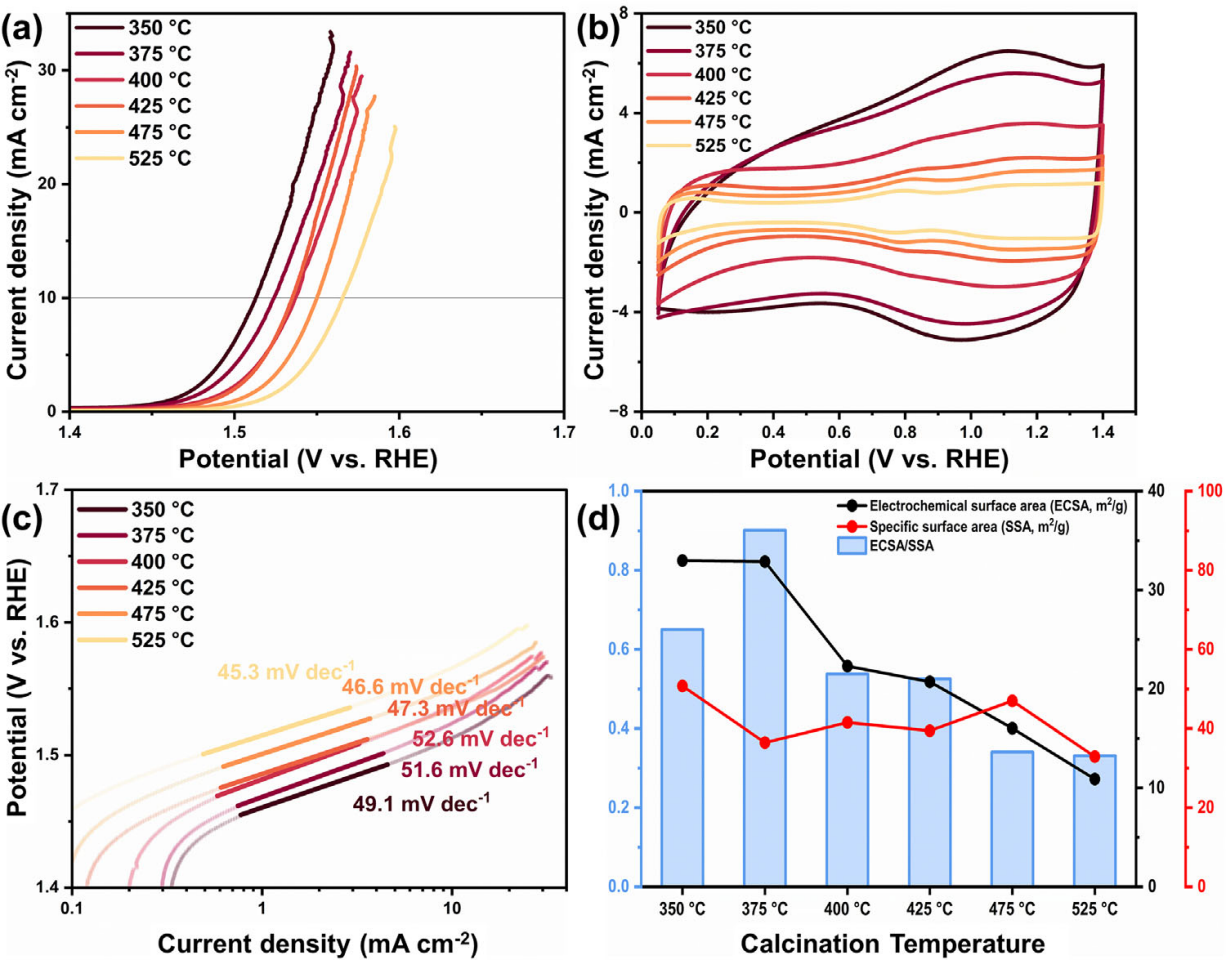
(a) Linear sweep voltammetry (LSV) curves of HDP‐IrO_x_ catalysts calcined at different temperatures, (b) Cyclic voltammetry (CV) curves, (c) Corresponding Tafel plots with calculated Tafel slopes, and (d) Comparison of electrochemical surface area (ECSA), specific surface area (SSA), and their ratio (ECSA/SSA) as a function of calcination temperature.

Electrochemical half‐cell measurements were performed on catalysts with different calcination temperatures. All measurements were conducted in 0.1 m HClO_4_ under N_2_ purging. The OER activity from the half‐cell measurements was evaluated by measuring the overpotential at 10 mA cm^−2^, which gradually increased with increasing heat treatment temperature. Overpotentials of 283, 295, 308, 306, 321, and 337 mV were observed for 350°C, 375°C, 400°C, 425°C, 475°C, and 525°C, respectively (Figure 4a). In addition, cyclic voltammetry (CV) measurements in the 0.05–1.4 V range confirmed that, with increasing calcination temperature, the CV curves became more distinct, with reduced Ir–OH species contribution, a clearer Ir^3+^/Ir^4+^ redox peak, and more pronounced redox behavior of IrO_2_ (Figure 4b).

Double‐layer capacitance (C_dl_) was obtained from CV measurements in the non‐faradaic region, and the electrochemically active surface area (ECSA) was calculated by dividing C_dl_ by the standard capacitance (C_s_). However, the calculated ECSA as listed in Table S1 was approximately 80 times larger than the measured SSA (Figure S12 and Table S1). This discrepancy arises because, in addition to the interfacial electrostatic charge accumulation represented by C_dl_, reversible redox reactions such as Ir^3+^/Ir^4+^ conversion and OH^−^ adsorption/desorption additionally contributed as pseudo‐capacitance [48].

Accordingly, electrochemical impedance spectroscopy (EIS) was applied to obtain the pure double‐layer capacitance of the catalysts [27, 49]. EIS was performed in potentiostatic EIS (PEIS) mode, and the measurement potential was set to 1.48–1.54 V near the OER onset potential. This condition was selected to analyze interfacial characteristics while minimizing the influence of overpotential accurately. EIS data were fitted using the equivalent circuit model shown in Figure S13 (R1, Q2‖R2, and Q3‖R3), allowing the separation of solution resistance, charge transfer resistance, and interfacial capacitive components. The calculated C_dl_ reflects interfacial characteristics while minimizing the influence of pseudo‐capacitance, and the ECSA obtained by this method more accurately reflects the actual ECSA than the CV‐based method. Consequently, with increasing calcination temperature, the ECSA decreased to 32.99, 32.88, 22.32, 20.73, 16.02, and 10.90 m^2^ g^−1^, respectively (Figure S14). This decrease is attributed to the conversion of the amorphous IrO_x_ into a rutile‐like structure at higher temperatures, which indicates a decrease in the amorphous contents.

In addition, the conductivity index [27, 40] was employed to evaluate the relative conductivity of the catalyst (Figure 4d). The conductivity index is defined as the ratio of ECSA to the SSA obtained from N_2_ adsorption–desorption isotherm measurements. According to the literature, metal oxide catalysts exhibit high electrochemical activity when the conductivity index exceeds unity. The catalyst calcined at 375°C exhibited the highest conductivity, with a conductivity index close to unity, compared to the catalyst calcined at 350°C and those prepared at other temperatures. This is attributed to the hybridization of two‐phase structures, in which amorphous and crystalline domains coexist, rather than to a purely amorphous catalyst. In particular, the large crystalline hollow structure acts as a conductive framework, providing electron pathways that facilitate efficient transport to the active sites of the amorphous region.

As a result, such a hybrid dual‐phase arrangement offers a new design strategy to mitigate the trade‐off between conductivity and OER activity. However, the formation of the dual‐phase structure is not solely determined by calcination temperature. Still, it is also strongly influenced by template size and surface chemistry, which govern the adsorption and diffusion behavior of Ir precursors. Based on this understanding, the structural evolution and electrochemical performance were further examined as a function of template size and the presence of PDA coating.

### 2.3 Effect of Polydopamine Coating on Electrochemical Performance

**FIGURE 5:**
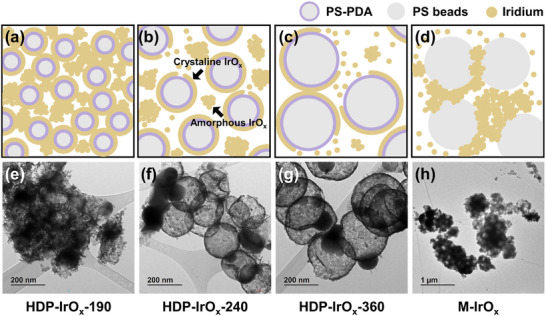
(a–d) Schematic illustration of the synthesis process before calcination, showing the formation of HDP‐IrO_x_ and M‐IrO_x_. (e–g) TEM images of HDP‐IrO_x_‐190, HDP‐IrO_x_‐240, and HDP‐IrO_x_‐360 catalysts synthesized with PS‐PDA templates of different diameters, and (h) TEM images of M‐IrO_x_ prepared with PS templates.

After confirming the effectiveness of the dual‐phase structure with PDA coating, it was further investigated whether a dual‐phase structure could be formed using PS beads without PDA coating. Catalysts were synthesized using templates of different sizes (190, 240, and 360 nm) with and without PDA coating, as illustrated in Figure 5a–d. The corresponding TEM images are presented in Figure 5e–h and Figure S15. The larger the size of the PDA‐coated template, the greater the number of binding sites available for fixing Ir ions. When the same amount of Ir precursor is used, more Ir ions are bound to catechol groups on larger templates, leaving fewer Ir ions in solution and thereby reducing the formation of amorphous IrO_x_. Conversely, smaller templates provide fewer catechol sites, leading to excess Ir ions in solution that precipitate as Ir(OH)_3_ under basic conditions, which consequently increase the amorphous IrO_x_ domain. For the 360 nm PS template, reducing the amount of Ir precursor did not result in hollow sphere formation (Figure S8). When the same precursor amount was used, the Ir layer thickness of the hollow sphere remained constant at approximately 6–7 nm. This indicates that the number of catechol binding sites in PDA is limited, and only a finite number of Ir ions can be fixed. Smaller templates, therefore, yield a higher fraction of amorphous IrO_x_, providing more active sites. In contrast, for the smallest template (HDP‐IrO_x_‐190), strong Ir aggregation occurred during calcination, leading to a loss of spherical morphology and the formation of agglomerated structures. Consequently, the HDP‐IrO_x_‐190 did not form a crystalline hollow shell structure.

**FIGURE 6:**
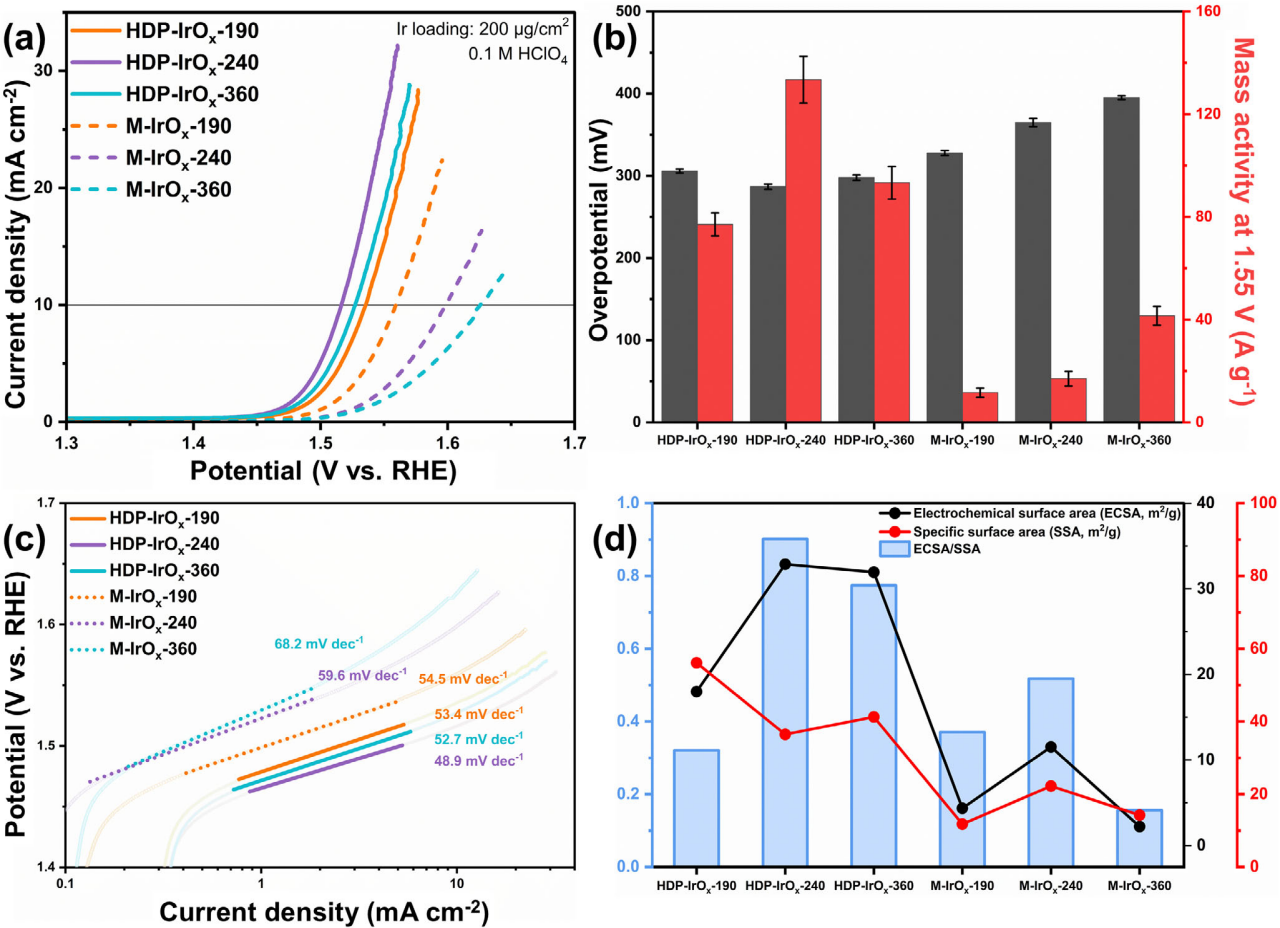
(a) LSV curves of HDP‐IrO_x_ and M‐IrO_x_ catalysts with different template sizes, (b) Comparison of overpotential and mass activity at 1.55 V (vs. RHE), (c) Corresponding Tafel plots with calculated Tafel slope, and (d) Comparison of ECSA, SSA, and c, onductivity index (ECSA/SSA) for HDP‐IrO_x_ and M‐IrO_x_ catalysts.

In the electrochemical measurements (Figure 6a), HDP‐IrO_x_‐190 without a hollow structure exhibited low activity, whereas HDP‐IrO_x_‐240 and HDP‐IrO_x_‐360 with hollow structures showed distinctly lower overpotentials. In particular, HDP‐IrO_x_‐240 exhibited the lowest overpotential (283 mV), which is less than 300 mV. This is attributed to the higher fraction of amorphous IrO_x_ formed in the smaller template, leading to a greater number of active sites. The highest mass activity was also observed for HDP‐IrO_x_‐240, reaching 133 A g^−1^ at 1.55 V versus RHE, owing to the increased amorphous IrO_x_ fraction and the resulting higher density of active sites (Figure 6b). In contrast, all M‐IrO_x_ catalysts existed as a single amorphous IrO_x_ phase, and larger template sizes resulted in higher overpotentials. In this case, the amorphous IrO_x_ single‐phase exhibits low conductivity and contributes insufficiently as active sites, suggesting the absence of conductive pathways provided by the crystalline phase. This interpretation is further supported by in ‐situ PEIS analysis conducted at OER‐relevant potentials (Figure S16), where HDP‐IrOx‐240 exhibited the smallest semicircle among the catalysts, indicating the lowest charge‐transfer resistance and faster reaction kinetics under operating conditions.

Notably, single‐phase amorphous IrO_x_ exhibited low activity, whereas the amorphous IrO_x_ within the crystalline domains effectively acted as active sites in the dual‐phase catalyst. This is interpreted as due to the crystalline hollow structure acting as a conductive framework that provides electron‐transfer pathways, thereby enabling the amorphous region to function as OER active sites. Accordingly, the hybrid dual‐phase structure exhibited enhanced electrochemical performance compared to the single‐phase amorphous catalyst.

Tafel slope analysis also confirmed this trend. HDP‐IrO_x_‐190, HDP‐IrO_x_‐240, and HDP‐IrO_x_‐360 with hybrid dual phases exhibited slopes of 53.4, 48.9, and 52.7 mV dec^−1^, respectively, while M‐IrO_x_‐190, M‐IrO_x_‐240, and M‐IrO_x_‐360, representing single‐phase amorphous catalysts, exhibited 54.5, 59.6, and 68.2 mV dec^−1^, respectively (Figure 6c). In general, dual‐phase catalysts typically yield slopes of 40–50 mV dec^−1^, whereas amorphous single‐phase catalysts yield slopes of about 60 mV dec^−1^, reflecting different rate‐determining steps (RDS) [50]. According to the literature, a slope of 60 mV dec^−1^ corresponds to the OH adsorption step as the RDS, while 40–50 mV dec^−1^ corresponds to the adsorption of –O– species as the RDS [51], which is consistent with the results of this study. Therefore, the presence or absence of PDA coating and the template size directly influence the formation of single‐ or dual‐phase structures, which in turn govern the OER activity.

It was confirmed that there was a clear difference in CV curves depending on the presence or absence of dopamine coating (Figure S17). In particular, the HDP‐IrO_x_ catalysts exhibited significantly higher ECSA values compared to the M‐IrO_x_ catalysts (Figure S18). The ECSA value for HDP‐IrO_x_‐240 was 32.88 m^2^ g^−1^, whereas that of M‐IrO_x_‐240 with the same template size was about three times lower at 11.53 m^2^ g^−1^. This difference arises because, in the single‐phase amorphous IrO_x_, the ECSA is low due to poor conductivity, whereas in the dual‐phase structure formed via PDA coating, hollow crystalline IrO_2_ provides a conductive framework, and amorphous IrO_x_ serves as active sites, with the two properties acting in a complementary manner.

The relative conductivity index also showed a similar trend (Figure 6d). The HDP‐IrO_x_ catalysts exhibited a significantly higher conductivity index than M‐IrO_x_, along with excellent electron transfer characteristics. In particular, HDP‐IrO_x_‐240 recorded the highest conductivity index despite having a relatively low SSA (36.46 m^2^ g^−1^), as shown in Figure S19. These results confirm that PDA coating not only increases the SSA but also optimizes electron‐transfer pathways and maximizes active site exposure.

The catalyst synthesized via PDA coating formed a dual‐phase structure with coexisting crystalline and amorphous domains, exhibiting excellent electrochemical activity through the cooperative action of conductive frameworks and active sites. In particular, when using 240 nm templates, a relatively large fraction of amorphous IrO_x_ was formed, increasing the density of active sites, thereby lowering the overpotential and improving mass activity. In contrast, in the absence of PDA coating, the catalyst existed as a single‐phase amorphous IrO_x_ and exhibited poor performance due to the lack of conductive pathways. Based on these half‐cell electrochemical analyses, a single‐cell test was conducted to evaluate the performance under actual PEMWE operating conditions.

### 2.4 PEMWE Single Cell Measurements

To confirm the performance of this catalyst under practical operating conditions, a single‐cell PEMWE test was conducted. An MEA was directly fabricated, with the cathode composed of a Pt‐coated gas diffusion layer (GDL, NARA Cell Tech, 0.3 mg_Pt_ cm^−2^, 40% Pt on Vulcan XC‐72). The anode catalyst was applied onto the membrane by spray coating, with loadings of 0.2 mg_Ir_ cm^−2^ (anode). A Nafion 115 membrane as a proton‐conducting membrane was employed.

**FIGURE 7:**
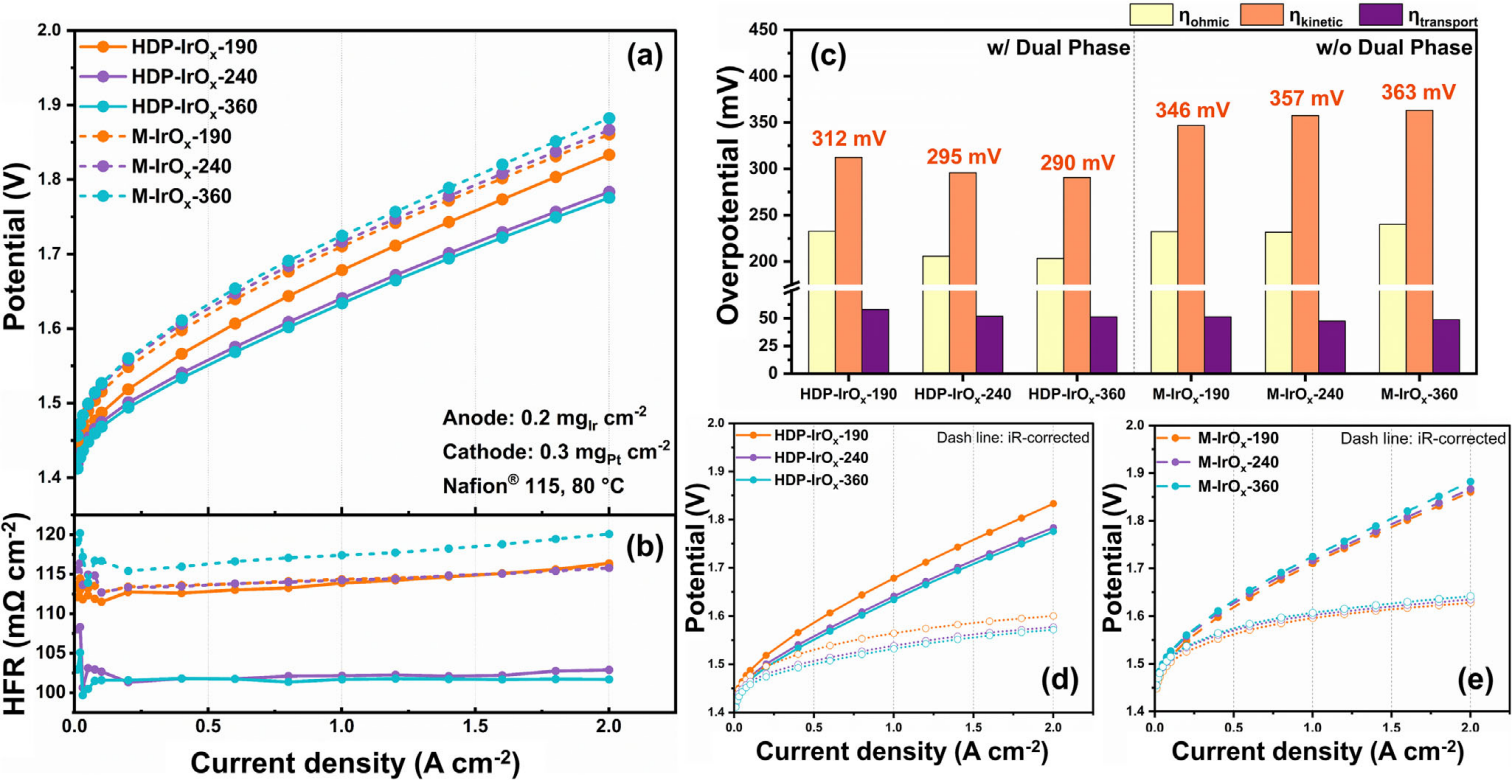
(a) Polarization curves of HDP‐IrO_x_ and M‐IrO_x_ catalysts with different template sizes, (b) Corresponding high‐frequency resistance (HFR) plots as a function of current density, (c) Comparison of overpotential components (ohmic, kinetic, and transport) for catalysts with and without dual‐phase structure at 2 A cm^−2^, and (d,e) polarization curves and HFR‐corrected polarization curves of (d) HDP‐IrO_x_ and (e) M‐IrO_x_ catalysts.

As shown in Figure 7a, HDP‐IrO_x_‐360 with the largest template size recorded 1.77 V at 2 A cm^−2^. For the catalysts HDP‐IrO_x_‐240, HDP‐IrO_x_‐190, M‐IrO_x_‐360, M‐IrO_x_‐240, and M‐IrO_x_‐190, the voltages recorded at 2 A cm^−2^ were 1.78, 1.83, 1.88, and 1.86 V, respectively. The performance of the HDP‐IrO_x_‐240 and HDP‐IrO_x_‐360 single cells with hollow‐structured dual‐phase catalysts showed no significant difference, leading to comparable performance. This confirmed that the presence of a hollow structure had a significant impact on performance in the practical MEA, whereas the difference in diameter had little effect. For comparison, the commercial IrO_2_ benchmark showed a cell voltage of 1.81 V at 2 A cm^−2^ (Figure S20).

The HFR is presented in Figure 7b. This corresponds to the pure resistance component, which is the sum of the electrolyte membrane resistance, the ionomer resistance, and the contact resistance. For the M‐IrO_x_ catalysts, HFR showed a gradual rise as the current density increased. This behavior was particularly evident for amorphous iridium oxide. In amorphous IrO_x_, the surface contains more OH^−^ species than in crystalline IrO_2_, resulting in higher hydrophilicity [13]. Due to this property, ionomers tend to adsorb more strongly, form thicker layers, or become locally aggregated. A continuous ionomer network is essential for proton transport; however, excessive adsorption or local aggregation disrupts this continuity, resulting in thinner and uneven conducting pathways. Therefore, as the current increases, the proton demand increases, but bottlenecks arise due to uneven conduction pathways, leading to decreased proton conductivity and increased HFR [52]. Thus, for the amorphous M‐IrO_x_ catalyst lacking crystallinity, HFR tended to increase at higher current densities. A similar trend was observed for the HDP‐IrO_x_‐240 catalyst. Although the increase was less pronounced than in M‐IrO_x_, it still exhibited a rising trend compared to HDP‐IrO_x_‐360. This is attributed to the HDP‐IrO_x_‐240 catalyst possessing a larger fraction of amorphous IrO_x_ sites than HDP‐IrO_x_‐360 (Figure S21). However, this increase was not clearly reflected in the polarization curves, resulting in no notable difference in polarization behavior, although a significant difference was observed in the durability results.

The overpotential analysis is presented in Figure 7c. The ohmic resistance values of the M‐IrO_x_ and HDP‐IrO_x_ catalysts were comparable, whereas the spherical dual‐phase catalysts HDP‐IrO_x_‐240 and HDP‐IrO_x_‐360 exhibited lower values of approximately 30 mV. The dual‐phase effect was also evident in the kinetic overpotential. The dual‐phase HDP‐IrO_x_ catalyst exhibited a low overpotential of 300 mV or less, whereas the single‐phase amorphous M‐IrO_x_ catalyst showed a higher overpotential of approximately 350 mV. These results demonstrate that the dual‐phase structure exhibits superior electrochemical performance compared to the single‐phase amorphous catalyst. However, for all of these catalysts, the mass transport overpotential was approximately 50 mV and remained nearly constant. This indicates that all catalysts possess hollow and porous structures, which facilitate water transport and oxygen bubble desorption.

**FIGURE 8:**
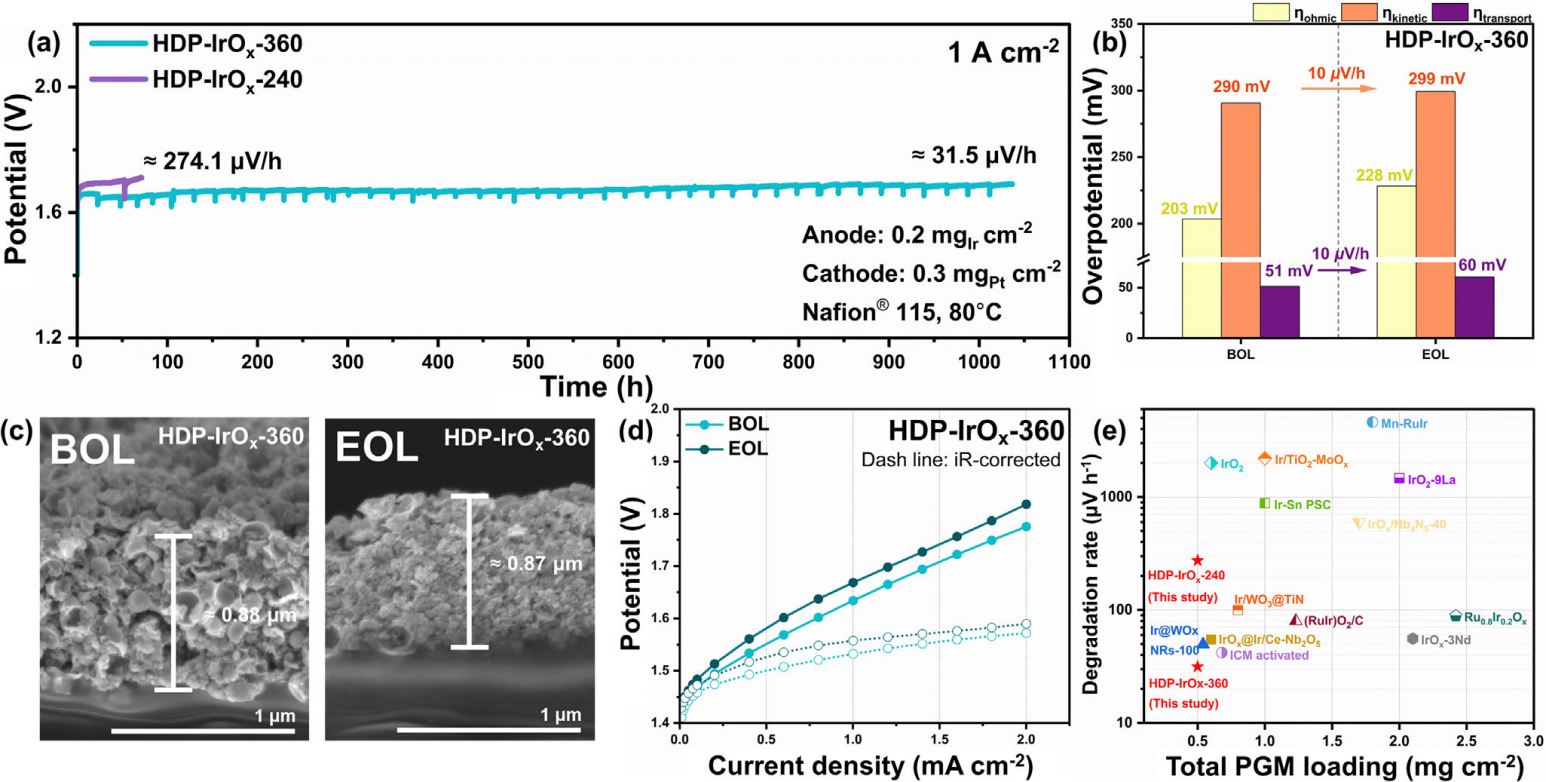
(a) Chronopotentiometry durability test of HDP‐IrO_x_‐240 and HDP‐IrO_x_‐360 catalysts at 1 A cm^−2^, (b) Comparison of overpotential components (ohmic, kinetic, and transport) BOL, and after EOL test for HDP‐IrO_x_‐360 at 2 A cm^−2^, (c) Cross sectional SEM images of HDP‐IrO_x_‐360 anodes at BOL and EOL, (d) Comparison of iR‐corrected polarization curves of HDP‐IrO_x_‐360 at BOL and EOL, and (e) Comparison of the total PGM loading and degradation rate for HDP‐IrO_x_‐360, HDP‐IrO_x_‐240 and Ir based catalysts in various states reported in previous reports.

The durability test was conducted at a constant current density of 1 A cm^−2^ with the same Ir loading (Figure 8a). The HDP‐IrO_x_‐240 MEA exhibited a degradation rate of 274.1 µV h^−1^ within 100 h. In contrast, the HDP‐IrO_x_‐360 MEA maintained a stable voltage for nearly 1036 h, with a much lower decay rate of 31.5 µV h^−1^. The decreased content of amorphous Iridium oxide domains resulting from the use of a larger template led to a decline in proton transport homogeneity. Consequently, the higher degradation rate of HDP‐IrO_x_‐240 is attributed to continuous proton accumulation arising from heterogeneous ionomer pathways. Furthermore, comparison with commercial IrO_2_ in a 100 h single‐cell durability test showed degradation rates of −6.0 µV h^−1^ for HDP‐IrO_x_‐360 and 64.4 µV h^−1^ for commercial IrO_2_, indicating the enhanced voltage stability of HDP‐IrO_x_‐360 (Figure S22).

Voltage loss (overpotential) analysis was conducted by separating overpotential components before and after the durability test at 2 A cm^−2^ (Figure 8b). For the HDP‐IrO_x_‐360 catalyst, the kinetic overpotential increased only by 10 µV h^−1^, and the mass transport overpotential exhibited a similar increase of 10 µV h^−1^. This remarkable durability arises from the synergistic effect of the dual‐phase hybridization in the hollow HDP‐IrO_x_ catalyst. The amorphous domains provide abundant active sites for the OER, while the crystalline Ir domains construct a robust electron conduction framework that effectively suppresses charge transfer resistance and the loss of active sites during long‐term operation. Furthermore, the hollow structure mitigates mass transport resistance, thereby enhancing durability. When compared with various states of iridium‐based catalysts reported in previous studies, the HDP‐IrO_x_‐360 proposed in this study exhibits the lowest PGM loading with a remarkably low degradation rate (Figure 8e, and Table S2). As observed in the cross‐sectional SEM images (Figure 8c), the thickness of the HDP‐IrO_x_‐360 catalyst layer remained unchanged after the extended durability test. These results confirm the excellent mechanical stability of the electrode under prolonged, harsh operating conditions.

Based on the overpotential separation analysis (Figure S23), the kinetic overpotential of HDP‐IrO_x_‐240 slightly decreased after the durability test, whereas the mass transport overpotential exhibited a substantial increase. Although the dual‐phase nature was retained, the loss of the hollow architecture reduced durability. This degradation behavior was also evident from the polarization curve (Figure S24), where the iR‐compensated voltage appeared higher due to the pronounced mass transport overpotential.

An economic assessment was also conducted for the MEA fabricated with the HDP‐IrO_x_‐360 catalyst. The Ir loading on the anode was 0.2 mg cm^−2^, corresponding to a precious‐metal cost of only 0.0289$ cm^−2^, and for the cathode, a Pt loading of 0.3 mg cm^−2^ corresponded to a cost of 0.0155$ cm^−2^. The total precious‐metal cost required to construct the MEA is therefore 0.0444$ cm^−2^. The energy efficiencies at 1 and 2 A cm^−2^ were 75.5% and 69.5%, respectively, with associated energy consumptions of 43.68 kWh kg^−1^ H_2_ and 47.57 kWh kg^−1^ H_2_. Based on the DOE calculation [53], the cost of producing 1 kg of H_2_ using this MEA was estimated to be 0.917$, which is sightly below the DOE ultimate target of 1$ kg^−1^. These results collectively highlight the excellent economic viability of the HDP‐IrO_x_‐360 catalyst. However, the reaction mechanisms occurring at the dual‐phase interface have not yet been fully clarified. Further mechanistic investigations into interfacial reaction pathways and structural evolution will be essential, providing a critical foundation for optimizing future catalyst design strategies and enhancing their applicability in practical PEMWE systems.

## 3 Conclusion

In summary, a dual‐phase hollow IrO_x_ catalyst was prepared via hard‐template‐assisted synthesis to overcome the intrinsic trade‐off between OER activity and durability. The complementary synergy between amorphous IrO_x_ and hollow crystalline IrO_2_ was found to enhance active‐site accessibility while simultaneously ensuring structural robustness. Furthermore, the influence of calcination temperature on dual‐phase formation and its excellence over single‐phase amorphous IrO_x_ was demonstrated. Variations in the distribution of amorphous domains with template size were also shown to affect catalyst durability and electrochemical performance directly. The MEA with HDP‐IrO_x_‐360 catalyst demonstrated the superior durability under harsh operation conditions over 1000 h, with a decay rate of only 31.5 µV h^−1^.

These results highlight that the synergistic cooperation between amorphous and crystalline domains plays an important role in facilitating charge transport and mitigating structural degradation during operation. Moreover, the phase‐engineering strategy presented here provides a generalizable design concept that can be extended to various metal oxide OER catalysts beyond Ir‐based systems. This dual‐phase design paradigm offers a promising route to developing next‐generation, highly efficient OER catalysts that reduce the amount of precious metals while enhancing scalability for practical PEMWE systems.

## Funding

This research was supported by the National Research Foundation of Korea (NRF) grant funded by the Korea government (MSIT) (No. RS‐2025‐02316898).

## Conflicts of Interest

The authors declare no conflicts of interest.

## Supporting information




**Supporting File**: advs74543‐sup‐0001‐SuppMat.docx

## Data Availability

The data that support the findings of this study are available from the corresponding author upon reasonable request.
