# Supplementary material for: Highly Robust Membrane Electrode Assembly Using Engineered Dual‐Phase Iridium Oxide Catalysts Breaking the Activity–Durability Trade‐Off in Proton Exchange Membrane Water Electrolysis
Source: Adv Sci (Weinh). 2026 Mar 10;13(25):e24243. doi: 10.1002/advs.202524243 (PMC13137850; doi:10.1002/advs.202524243)
Supplement: Supplementary file 1 — Supporting File: advs74543‐sup‐0001‐SuppMat.docx [file ADVS-13-e24243-s001.docx]

Supporting Information

Highly Robust Membrane Electrode Assembly using Engineered Dual-Phase Iridium Oxide Catalysts Breaking the Activity–Durability Trade-Off in Proton Exchange Membrane Water Electrolysis

Ho Seong Yang^1^, Song Gyun Kim^1^, Rubin Shin^1^, Youngin Cho^1^, and Chanho Pak^1,2*^

Ho Seong Yang^1^, Song Gyun Kim^1^, Rubin Shin^1^, Youngin Cho^1^, and Chanho Pak^1,2^

^1^Graduate School of Energy Convergence, Gwangju Institute of Science and Technology, Gwangju, Republic of Korea

^2^Department of Chemistry, Gwangju Institute of Science and Technology, Gwangju, Republic of Korea
E-mail: chanho.pak@gist.ac.kr

**Experimental Section**

*Synthesis and surface modification of polystyrene beads*

First, polystyrene (PS) beads were synthesized through the emulsion polymerization method. 0.1 g of sodium dodecyl sulfate (SDS, >99%, Sigma-Aldrich) was dissolved in 100 mL of deionized water (DIW) and stirred at 70 °C for 30 min. Afterward, 10 mL of styrene monomer (>99%, Sigma-Aldrich) was added, followed by 0.2 g potassium persulfate (KPS, >99%, Sigma-Aldrich). The mixture was refluxed at 70 °C for 24 h to complete the polymerization.

Subsequently, a polydopamine (PDA) coating was formed on the PS beads by a self-polymerization process. One gram of PS beads was dispersed in a dopamine hydrochloride solution (>99%, Alfa Aesar), and 100 mL of 10 mM tris-hydrochloride (Tris-HCl, pH 8.5, Biosesang) solution was added. The pH was adjusted to 8.5 with 0.2 M sodium hydroxide solution (NaOH, >85%, Duksan). The suspension was stirred for 40 min at room temperature in the dark. After the reaction, the product was collected by centrifugation, and the supernatant was removed.

*Preparation of iridium oxide catalyst*

To synthesize hollow dual-phase iridium oxide (HDP-IrO_x_), a hard-template method was employed. This was followed by an iridium immobilization process. PDA-coated PS beads were dispersed in 20 mL of DIW. Then, 0.2 g of iridium (III) chloride (Ir 54.08 %, T&I Chem) was dissolved in 25 mL of DIW under continuous stirring for 1 h. The two solutions were subsequently mixed. The pH of the mixed suspension was adjusted to 9 using an NH_3_/NH_4_Cl buffer solution prepared from ammonium hydroxide (28%-30%, Junsei Chemical) and ammonium chloride (>98%, Sigma-Aldrich). The resulting mixture was refluxed at 80 °C for 3 h. After cooling to room temperature, the product was filtered and dried overnight in an oven. To remove the hard template, the dried composite was calcined in air at different high temperatures in the range of 350 to 500 ℃ for 1 h. The heating rate was carefully maintained at 1 ℃ min^-1^ to prevent iridium agglomeration. For comparison, microporous iridium oxide (M-IrO_x_) was synthesized under identical conditions using PDA-free PS beads as the template.

*Material characterization*

The particle size and zeta potential of the synthesized PS beads and PDA-coated PS were measured using dynamic light scattering (DLS, Zetasizer Nano ZSP, Malvern Instruments). The successful coating of PDA on the PS beads was confirmed by Fourier-transform infrared spectroscopy (FT-IR, Vertex 70v, Bruker) and Raman spectroscopy (LabRAM HR Evolution, Horiba). The morphological characteristics of the prepared catalysts were examined by scanning transmission electron microscopy (STEM, Tecnai G2 F30 S-Twin, FEI) and scanning electron microscopy (SEM, Apreo 2 S LoVac, Thermo Fisher Scientific). Atomic-resolution TEM and STEM analyses were performed using a double Cs-corrected transmission electron microscope(Cs-TEM, JEM-ARM300F2, JEOL). The elemental distribution was analyzed using energy-dispersive X-ray spectroscopy (EDS). The specific surface area was estimated by BET equation using nitrogen adsorption–desorption isotherms (BELSORP MAX volumetric adsorption analyzer, MicrotracBEL). The surface chemical composition and oxidation states were investigated by X-ray photoelectron spectroscopy (XPS, K-Alpha+, NEXSA, Thermo Fisher Scientific). The XPS data were analyzed using the CasaXPS software. The background was fitted using Shirley function, and the main C 1s peak was calibrated to 284.48 eV prior to spectral fitting. The raw spectra were fitted using Gaussian/Lorentzian (GL) and Lorentzian Asymmetric (LA) line shapes. The Ir 4f _7/2_ and Ir 4f _5/2_ peaks were separated by 3.0 eV with a peak area ratio of 4:3. The O 1s spectra were fitted using three components corresponding to different Ir–O coordination environments, namely μ_1_-O, μ_2_-O, and μ_3_-O species. The binding energy shift from μ_1_ to μ_3_ reflects an increase in Ir-O coordination number and associated electron density on oxygen (**Figure S9**), consistent with literature-reported IrO_x_ systems^[1]^. The crystalline structure was characterized using a high-resolution powder X-ray diffractometer (XRD, Cu Kα, SmartLab, Rigaku).

*Electrochemical measurements*

Electrochemical measurements were performed using a potentiostat (VSP-150, BioLogic) in a three-electrode configuration at 25 ℃ in 0.1 M HClO_4_ (70%, Daejung). A glassy carbon rotating disk electrode (RDE, 5 mm in diameter) was used as the working electrode, with a reversible hydrogen electrode (RHE, Gaskatel) and a platinum wire serving as the reference and counter electrodes, respectively. The catalyst ink was prepared by mixing 1.48 mL of DIW and 0.5 mL of isopropyl alcohol (volume ratio 3:1), followed by the addition of Nafion^®^ solution as a binder. The catalyst loading on the electrode surface was maintained at 200 µg cm^-2^ for all samples. Before each electrochemical measurement, the electrode was activated through 20 cyclic voltammetry (CV) cycles within a potential window of 0.05–1.4 V. Linear sweep voltammetry (LSV) was then conducted from 1.2 V to 1.7 V at a scan rate of 5 mV s^-1^ under 1600 rpm rotation. Each experiment was repeated five times to ensure reproducibility. iR-compensation was applied at 85%, based on the uncompensated resistance obtained from impedance measurements.

The Tafel slope was determined according to the following equation (1):

$\eta=a+b\log j$ (1)

Where b denotes the slope of the fitted line. The electrochemical surface area (ECSA) was calculated from the double-layer capacitance (C_dl_) using two complementary methods: (i) CV measurements at various scan rates and (ii) potential electrochemical impedance spectroscopy (PEIS). For the first method, CVs were obtained in the non-faradaic region (1.0–1.15 V), and ECSA was derived by dividing C_dl_ by the specific capacitance^[2]^ (C_s_ = 0.035 mF cm^-2^). In the second method, PEIS measurements were performed between 1.48 V and 1.54 V, with a 15 mV amplitude, over the frequency range of 10 mHz to 100 kHz, following the procedure reported in a previous study.^[3]^

*PEMWE measurements*

The membrane electrode assembly (MEA) was fabricated using a Nafion^®^ 115 membrane. The anode employed the synthesized catalyst, whereas the cathode consisted of a commercial Pt/C-coated carbon paper (NARA Cell Tech, 0.3 mg_Pt_ cm^-2^, 40 wt% Pt on Vulcan XC-72). Before coating, the Nafion^®^ membrane was sequentially washed in 30 wt% H_2_O_2_, deionized water, and 0.1 M H_2_SO_4_ at 80 ℃ for 1 h each. After drying, the anode catalyst layer was directly deposited onto the membrane to form a catalyst-coated membrane (CCM). A Pt-uniformly coated Ti felt (Bekaert) was employed as the porous transport layer (PTL). For the cathode electrode, a gas-diffusion electrode (GDE) coated with Pt/C (40 wt% Pt on Vulcan XC-72) was employed. The anode catalyst ink was prepared from deionized water, isopropyl alcohol, and 10 wt% Nafion^®^ ionomer solution. The catalyst content and ionomer content were fixed at 0.8 wt% and 10 wt%, respectively. The ink was ultrasonicated in an ice bath for 1 h to ensure complete dispersion. The catalyst loading on the anode was set to 0.2 mg_Ir_ cm^-2^, which was verified by X-ray fluorescence spectroscopy (XRF). The total active area of the MEA was 4 cm^2^. For comparison, a membrane electrode assembly (MEA) was fabricated using a commercial iridium oxide(99.99 %, Premion^®^, thermo scientific) as the anode catalyst, while maintaining the same total PGM loading. The PEMWE performance was evaluated at 80 ℃ using a potentiostat (VSP-150, BioLogic). The polarization curve was obtained through galvanostatic electrochemical impedance spectroscopy (GEIS) over a frequency range of 30 kHz to 100 MHz. The impedance spectra were recorded at current densities of 10, 20, 30, 50, 75, 100, 200, 400, 600, 800, 1000, 1200, 1600, 1800, and 2000 mA cm^-2^. The collected data were subsequently analyzed to separate the individual resistance components within the cell. The durability test was carried out at a current density of 1 A cm^-2^ using a power supply (PWX750LF, Kikusui).

The cell voltage (E_cell_) of PEMWE consists of four main components: the reversible cell potential (E_rev_) and three types of overpotentials, namely the ohmic overpotential (η_ohm_), the kinetic overpotential (η_kin_), and the mass transport overpotential (η_mass_). The ohmic overpotential can be expressed as the product of the measured current density and the internal resistance of the PEMWE, as shown in Equation (2).

$\eta_{ohm}=j(mA/{cm}^{2})\times HFR\left( \Omega\cdot{cm}^{2} \right)$ (2)

Furthermore, the hydrogen evolution reaction (HER) was assumed to be non-polarizable, and thus the overpotential associated with the reaction kinetics was considered to originate solely from the OER. The kinetic overpotential (η_kin_) was determined based on the slope (b) obtained from the Tafel equation, which is defined as follows (Equation 3):

$b=\frac{2.303\times R\times T}{4\times F}$ (3)

The obtained b value was then used to calculate the **kinetic overpotential (η_kin_)** according to Equation (4).

$\eta_{kin}=b\times\log(j/j_{0})$ (4)

The mass transport overpotential (η_mass_) was calculated by subtracting the reversible potential, the ohmic overpotential, and the kinetic overpotential from the total cell voltage, as expressed in Equation (5):

$\eta_{mass}=E_{cell}-(E_{rev}+\mu_{ohm}+\mu_{kin})$ (5)

*Cost and Energy Metrics Evaluation*

The cost of the noble metals used in the anode electrodes was obtained from the Johnsom Matthey Price Charts.^[4]^

The energy efficiency of the PEMWE system was calculated using the following equation (6):

$\text{Energy efficiency}= \frac{1.23 V}{U_{cell}}$ (6)

Here, 1.23 V represents the theoretical energy of the products, and *U_cell_* corresponds to the measured cell voltage at 1 A cm^-2^ and 2 A cm^-2^.

The energy consumption was determined according to equation (7):

$\text{Energy consumption}= \frac{U_{cell}\times I_{cell}\times t}{m_{H_{2}}}$ (7)

where *I_cell_* is the applied current (A), *t* is the operation time, and $m_{H_{2}}$ is the mass of hydrogen generated during *t*. The hydrogen mass was calculated by equation (8):

$m_{H_{2}}= \frac{I_{cell}\times t}{z\times F}\times M_{H_{2}}$ (8)

where *z* is the number of electrons required to produce hydrogen, *F* is the Faraday constant (96,485 C mol^-1^), and $M_{H_{2}}$is the molecular mass of hydrogen.

The hydrogen production cost was calculated as equation (9):

$\text{Cost = Energy consumption × Electricity bill}$ (9)

The electricity price was taken from previous reports (US$0.02 kWh^-1^).^[5]^


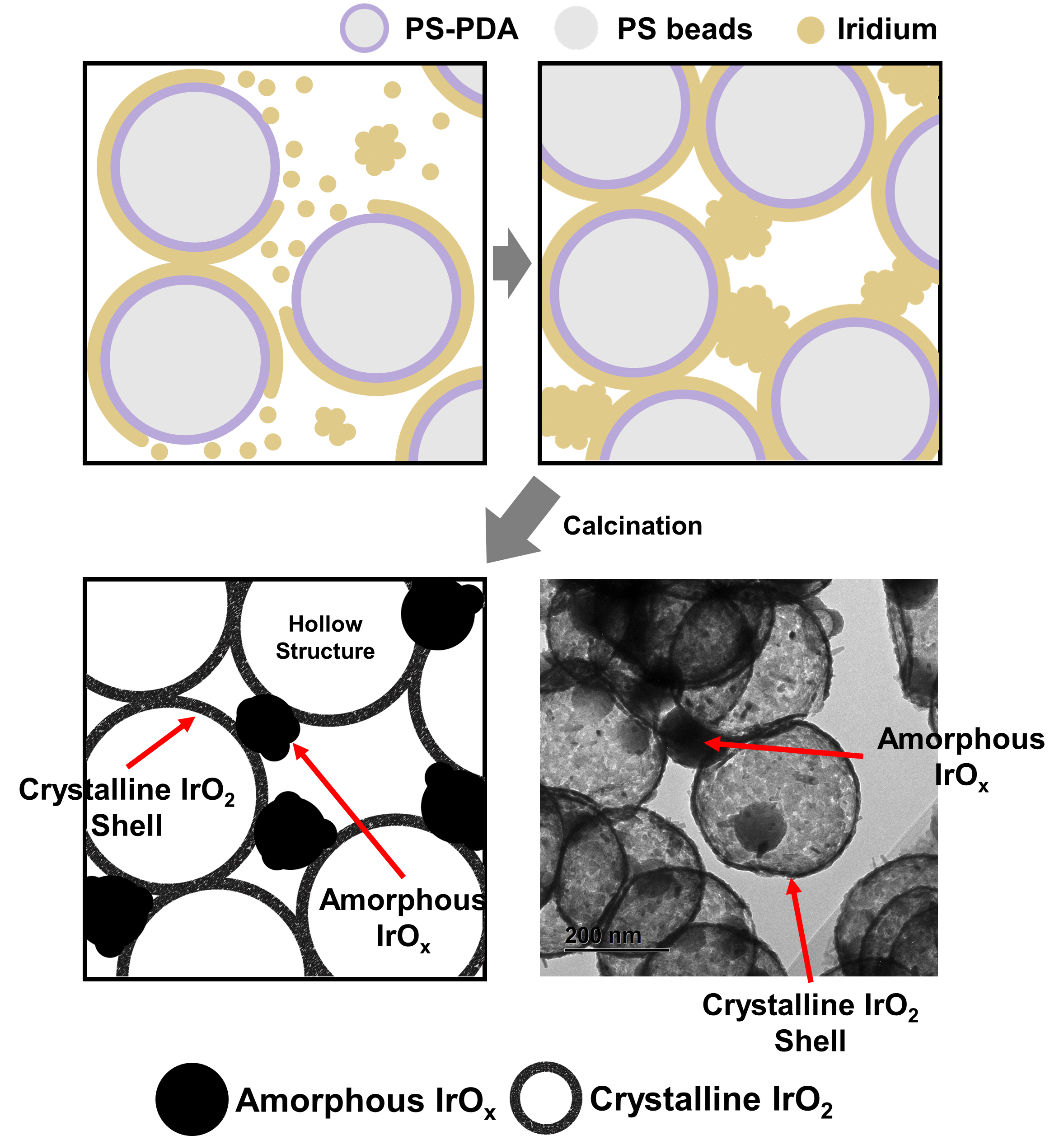


**Figure S1.** Schematic illustration of the spatial distribution of crystalline and amorphous IrO_x_ phases in the PS-templated dual-phase structure.


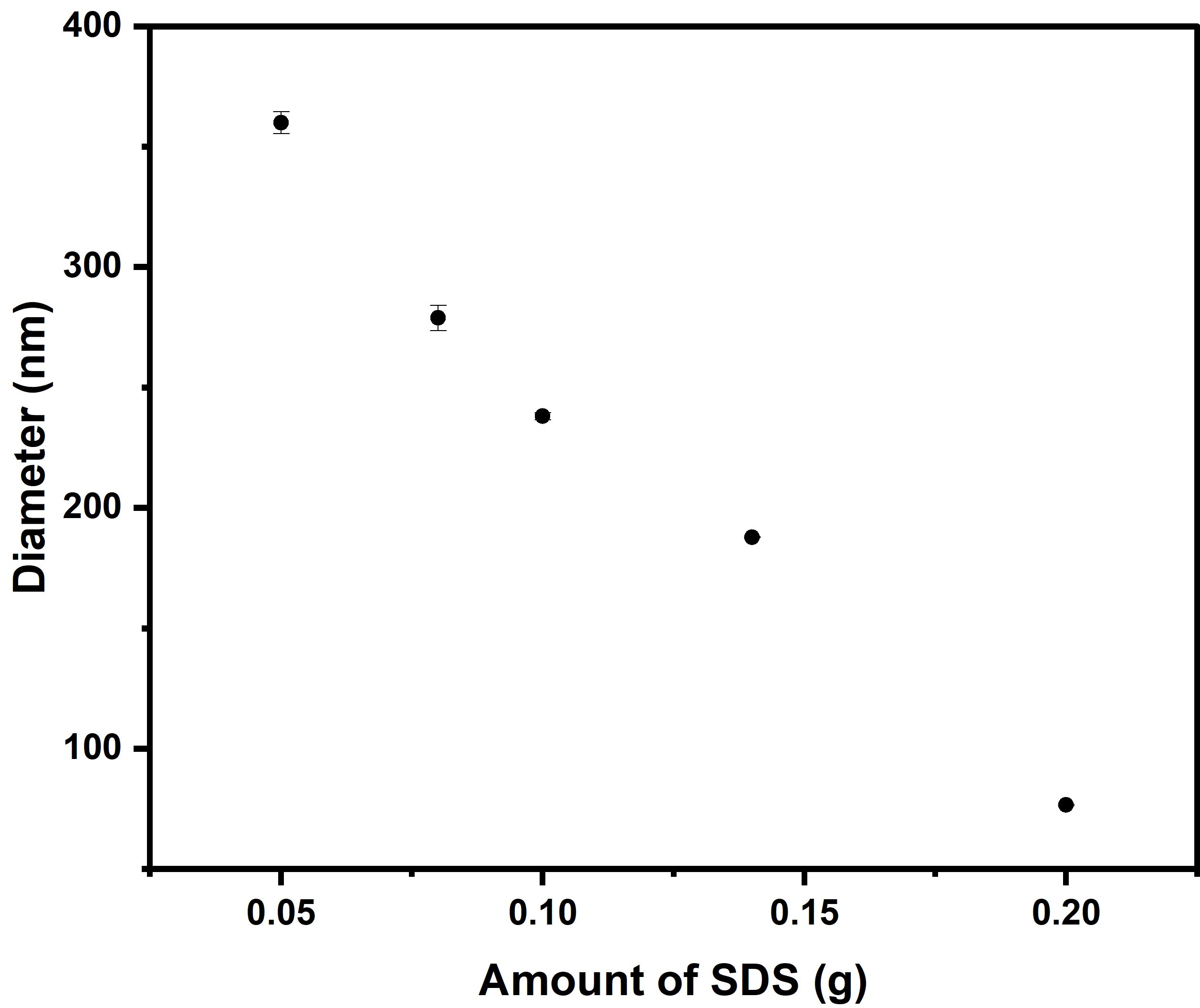


**Figure S2** Measurement of the diameter of polystyrene (PS) beads synthesized with varying amounts of sodium dodecyl sulfate (SDS) using dynamic light scattering (DLS).


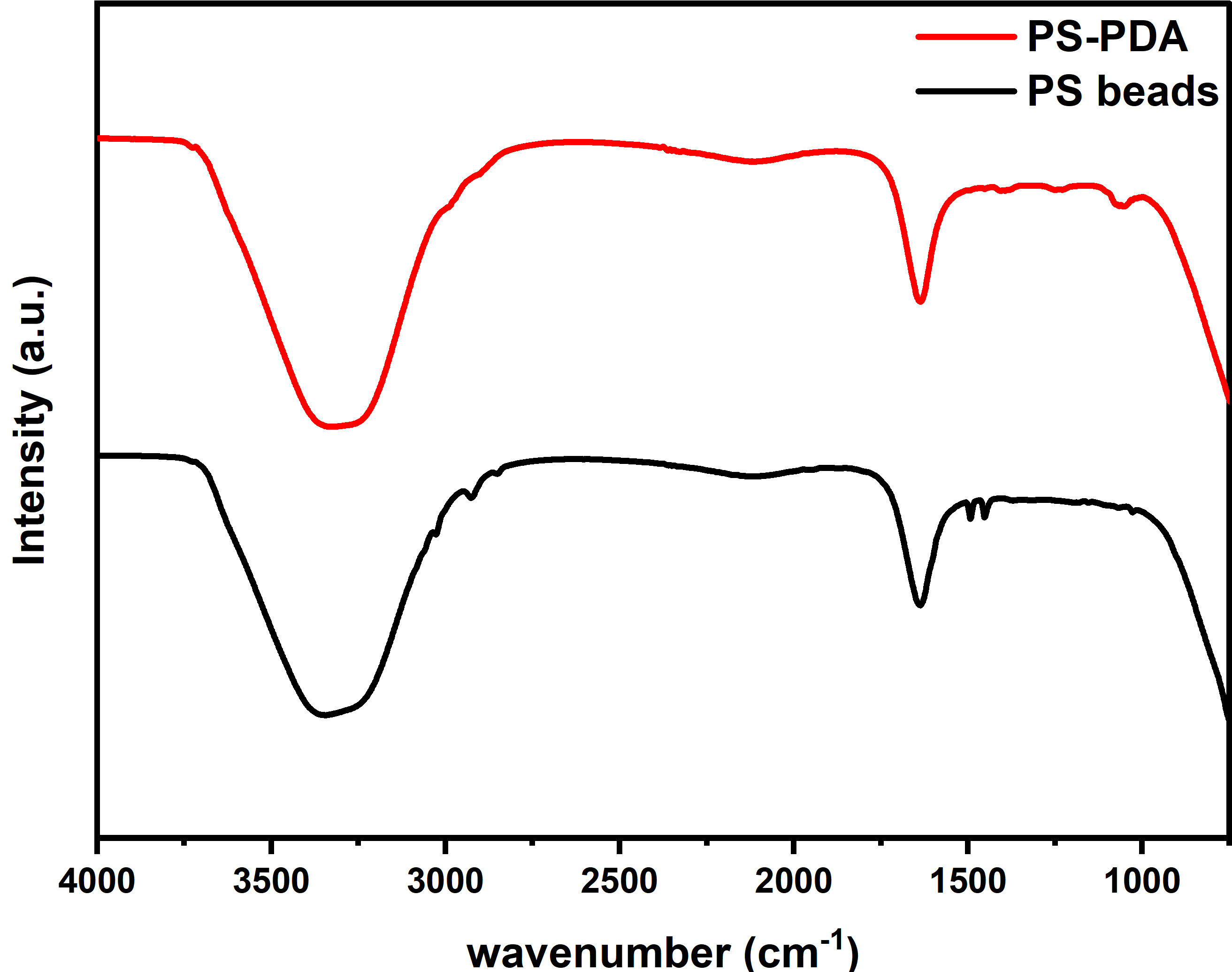


**Figure S3** Comparison of Fourier transform infrared (FT-IR) spectra of PS beads and PS–PDA composites.


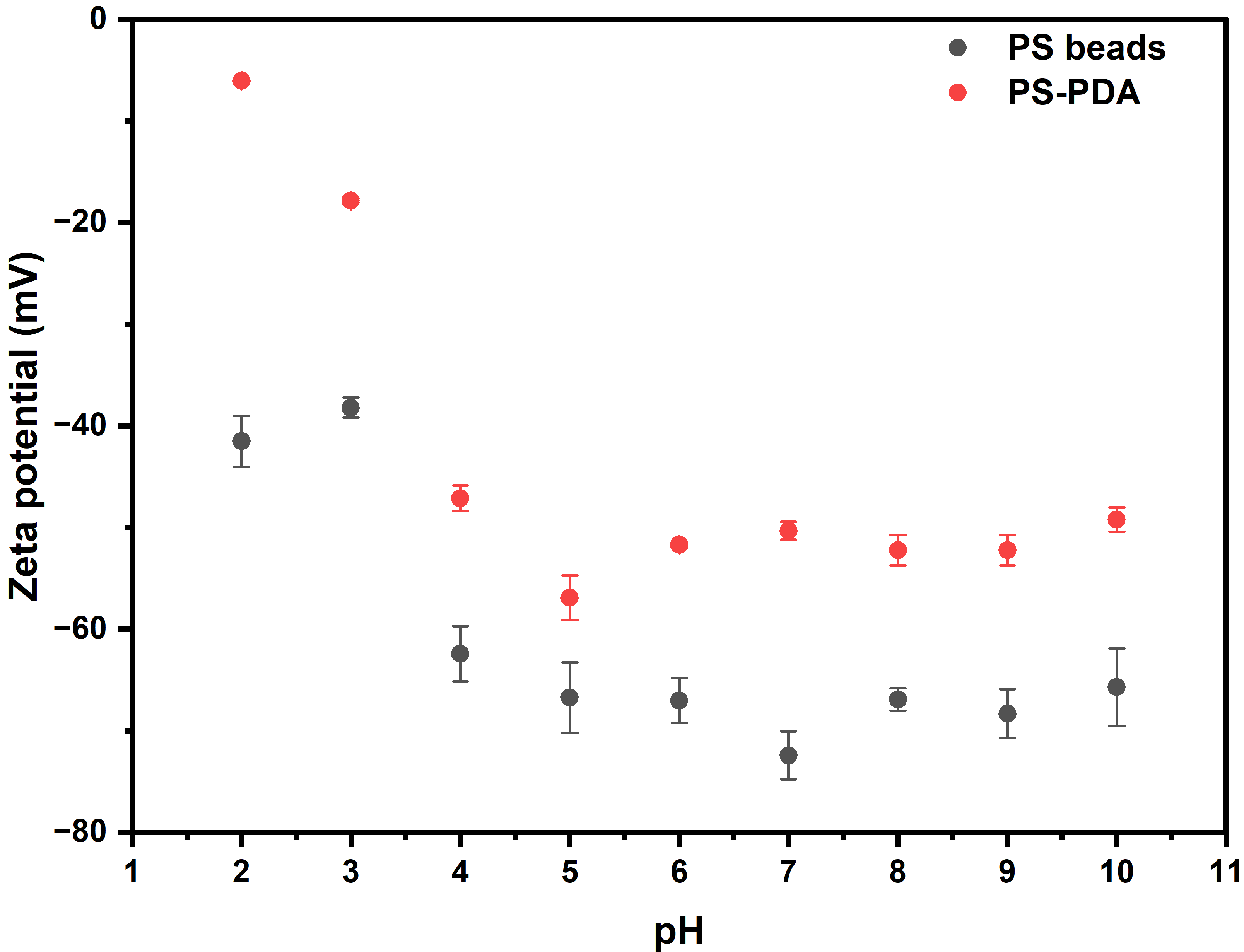


**Figure S4.** Comparison of zeta potentials of PS beads and PS–PDA composites as a function of pH.


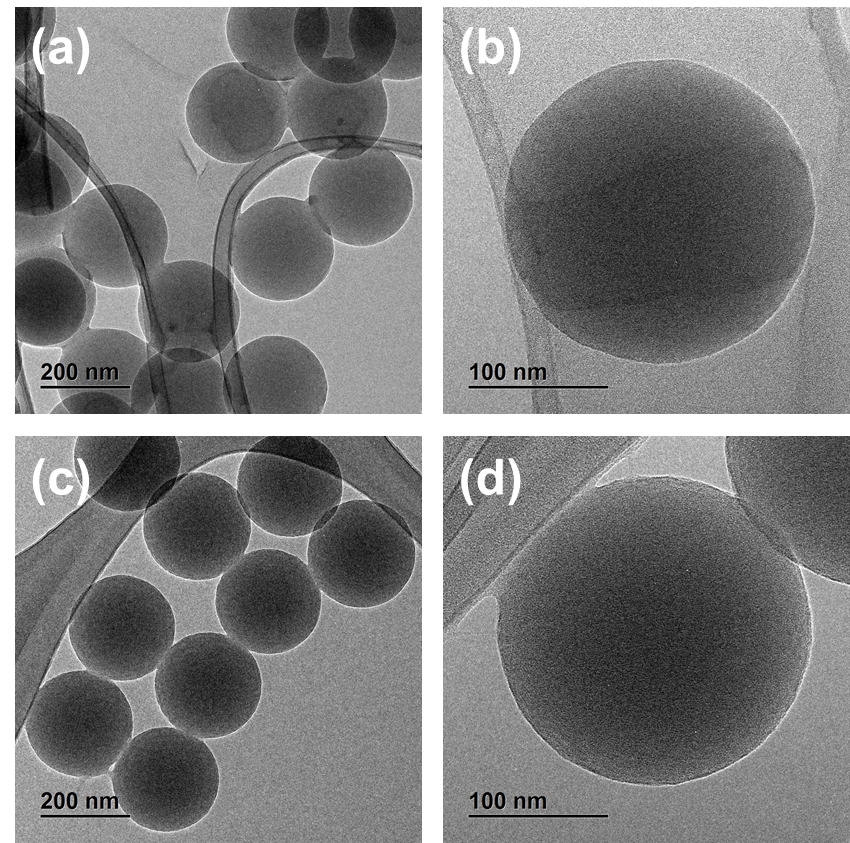


**Figure S5.** TEM analysis of (a, b) PS beads and (c, d) PS–PDA composites.


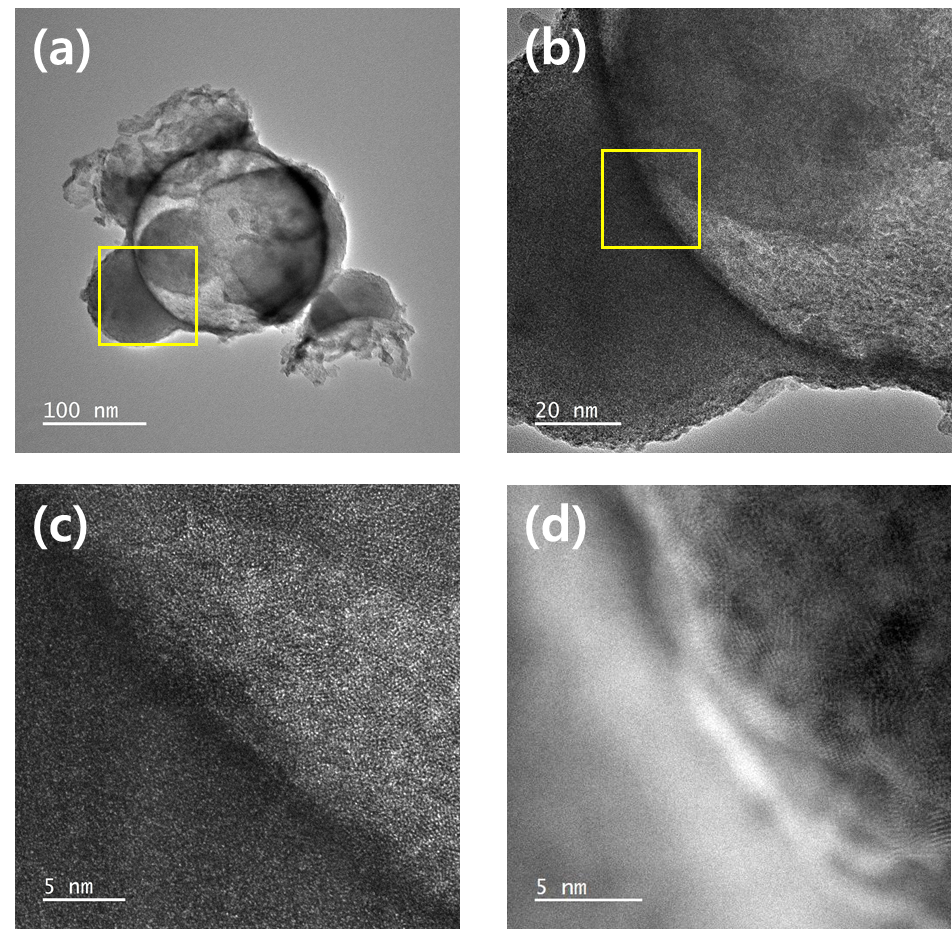


**Figure S6.** (a,b) Low- and high-magnification Cs-corrected transmission electron microscopy (Cs-corrected TEM) images of the hollow IrO_x_ particle. (c) Atomic-resolution Cs-corrected TEM image acquired from the highlighted region in (b), showing a featureless contrast without discernible lattice fringes, characteristic of the amorphous IrO_x_ domain. (d) Atomic-resolution scanning transmission electron microscopy (STEM) image obtained from the crystalline shell region, where clear lattice fringes are directly visualized, confirming the crystalline nature of the hollow IrO_2_ domain.


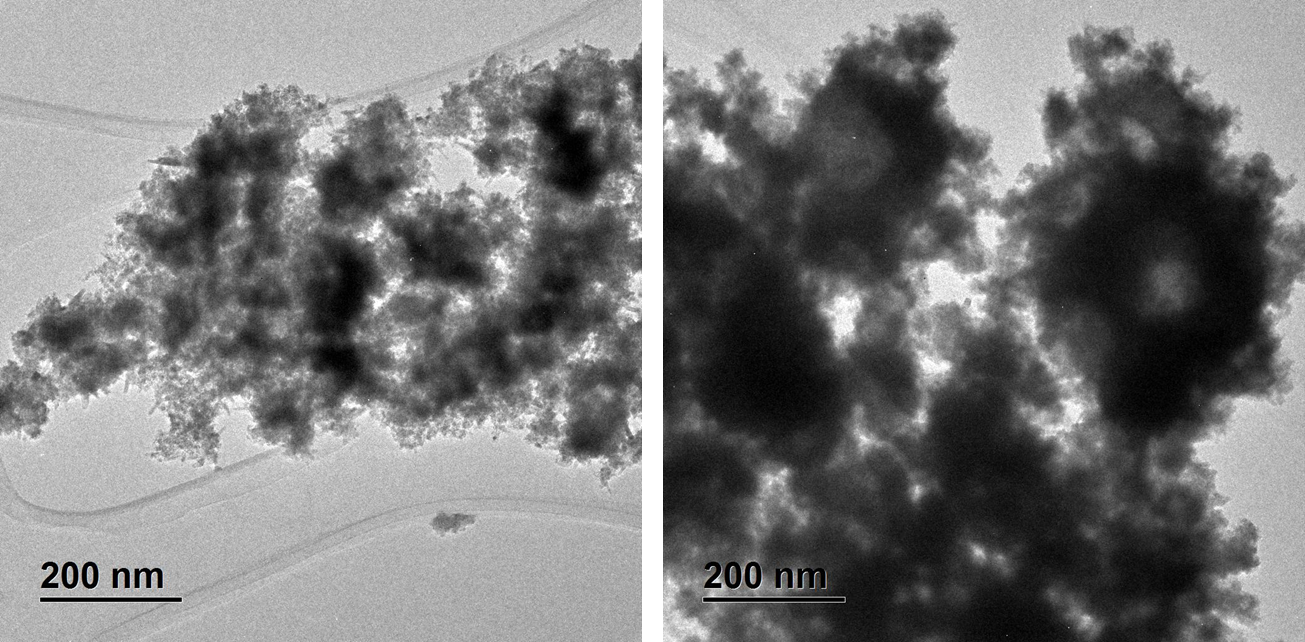


**Figure S7.** TEM analysis of the catalyst synthesized using PS beads as a template without PDA coating.


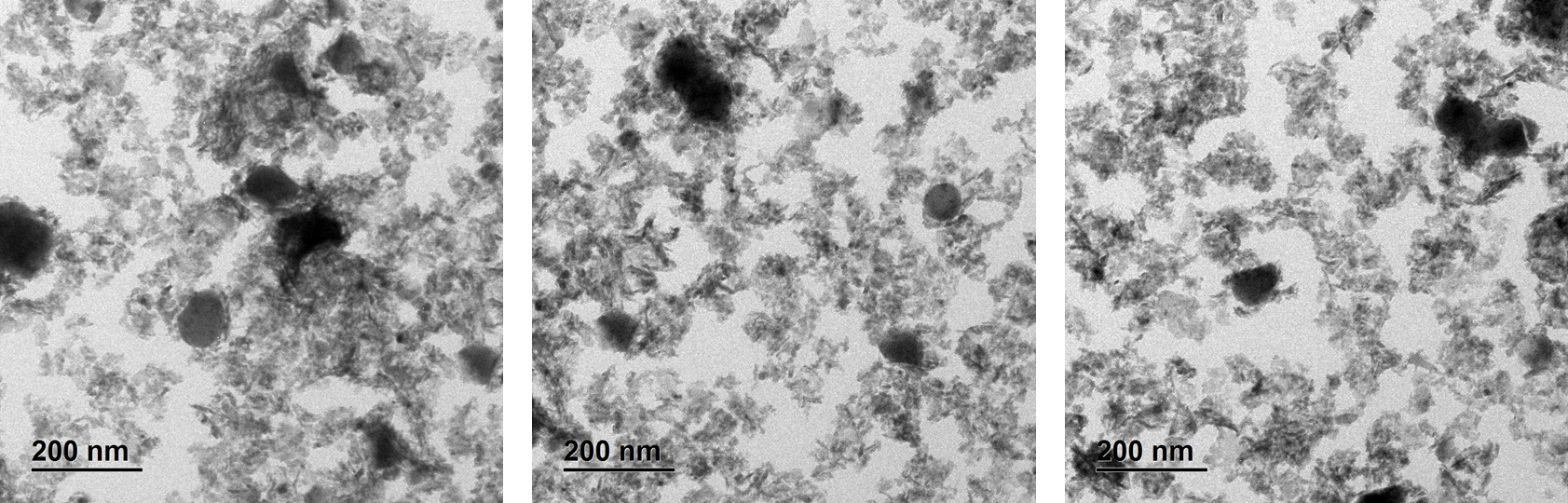


**Figure S8.** TEM analysis of the catalyst synthesized using a smaller amount of IrCl_3_ (0.1 g) compared to the conventional 0.2 g.


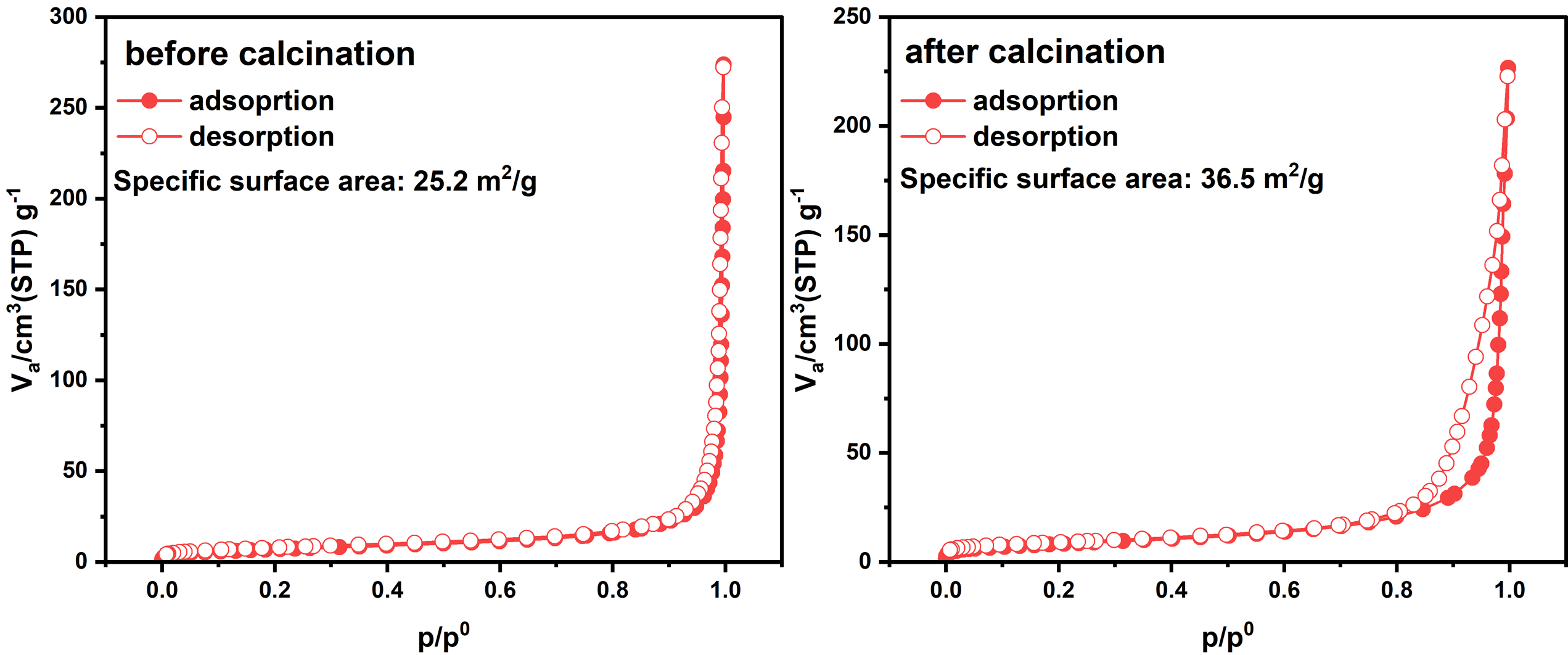


**Figure S9.** Comparison of nitrogen(N_2_) adsorption–desorption isotherms before and after the calcination process.


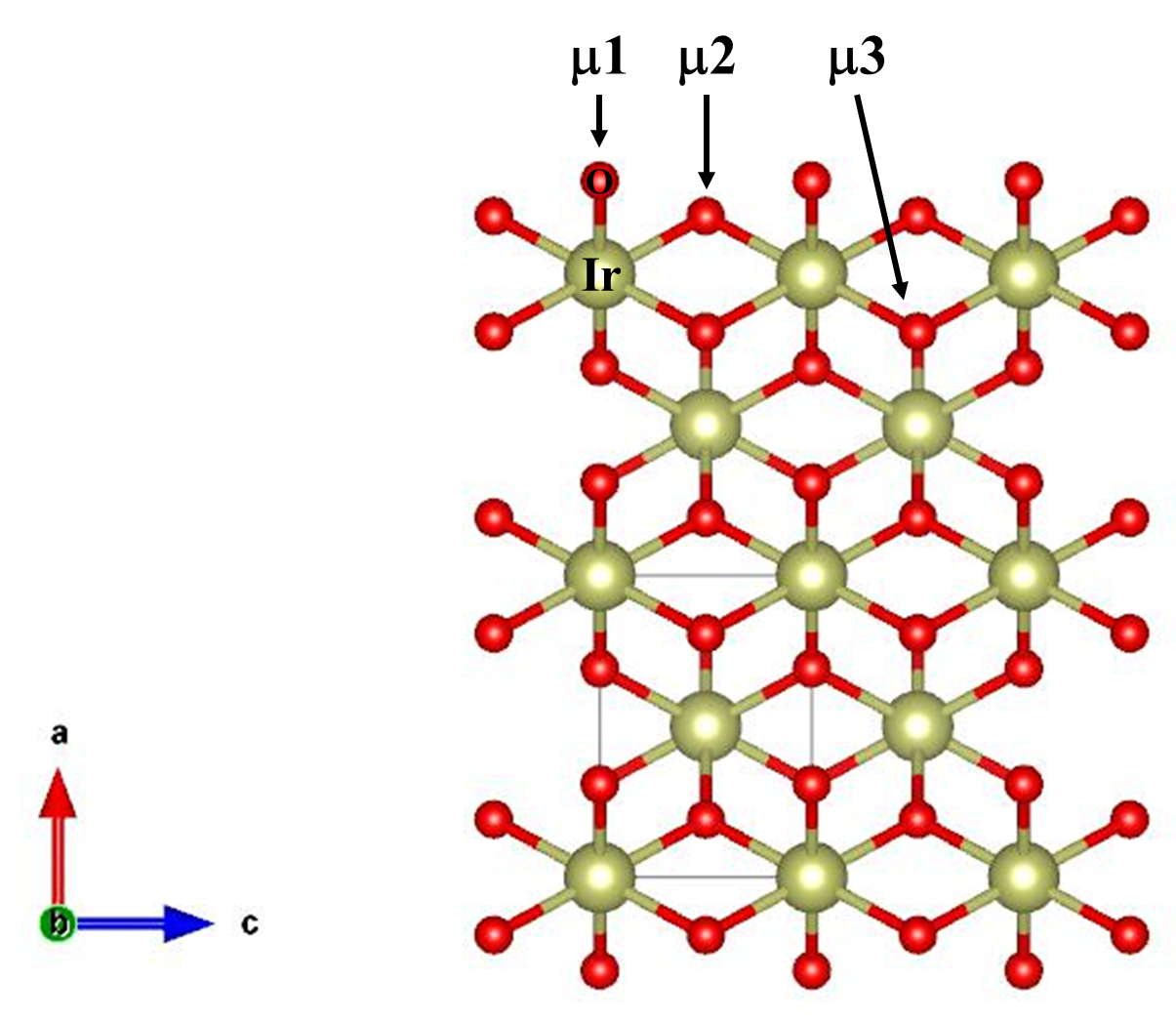


**Figure S10.** Schematic illustration of the rutile IrO_2_ crystal structure showing surface oxygen species with different coordination numbers: μ_1_, μ_2_, and μ_3_ represent oxygen atoms bonded to one, two, and three Ir atoms, respectively.


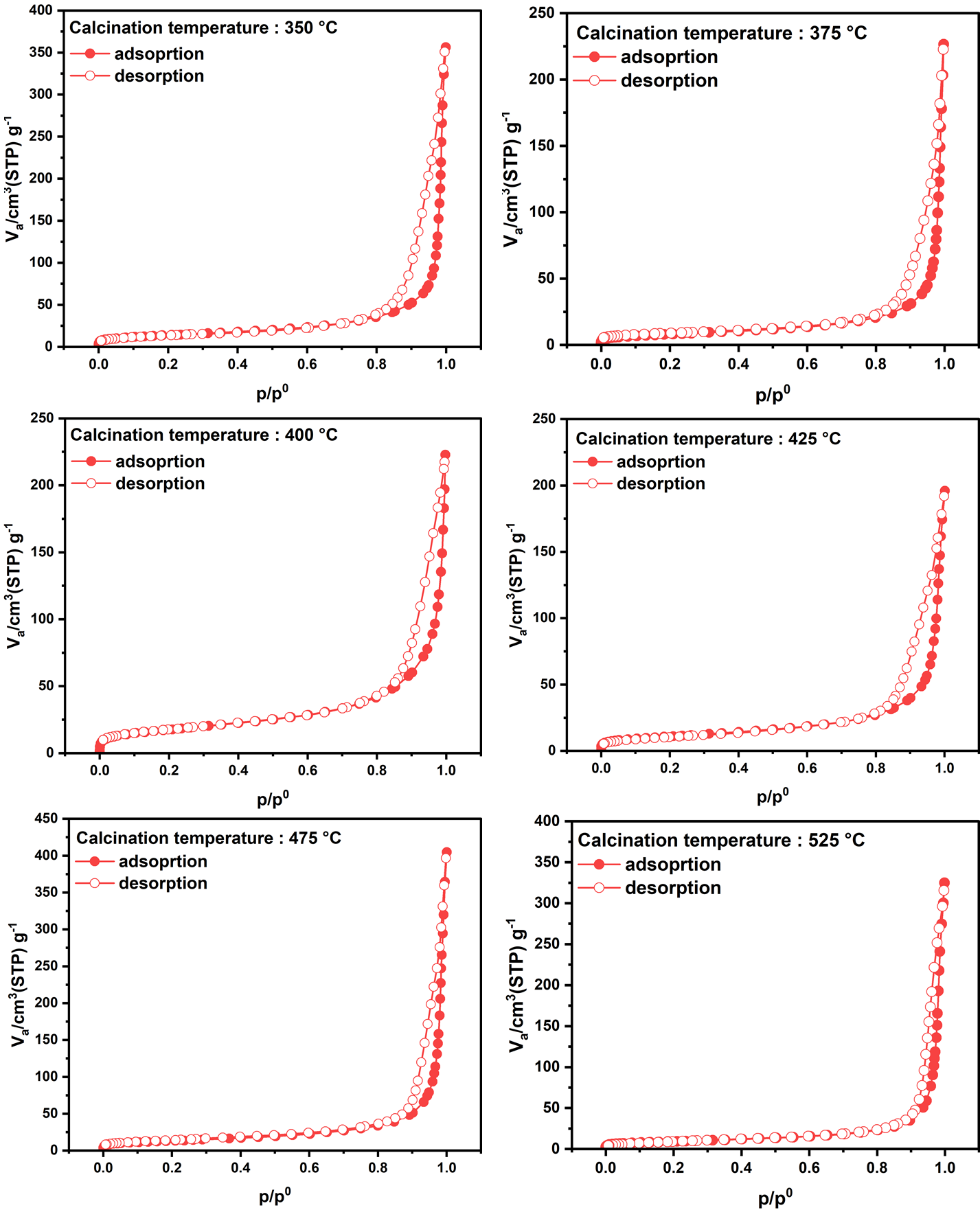


**Figure S11.** Comparison of nitrogen(N_2_) adsorption–desorption isotherms obtained under different calcination temperature conditions.


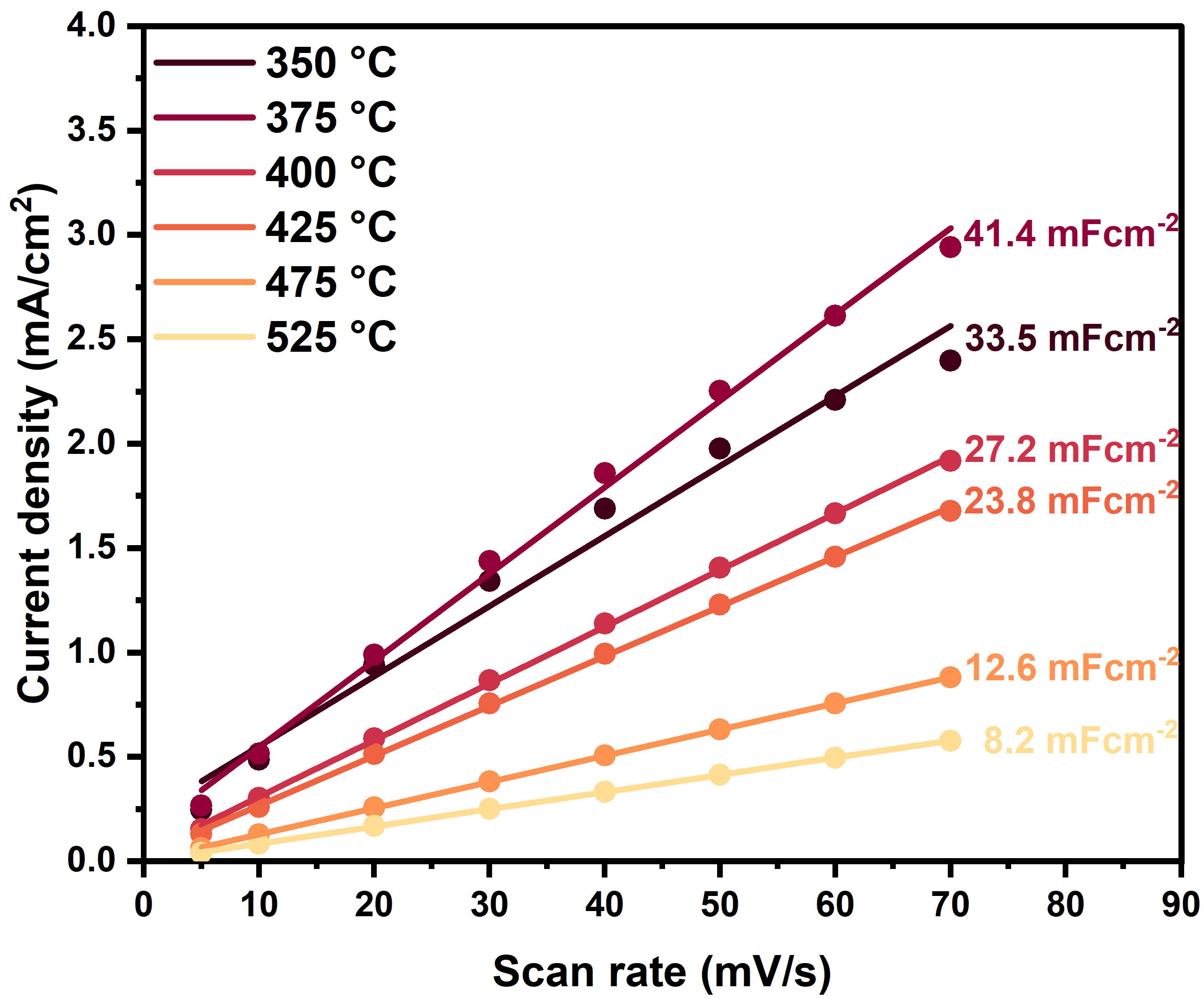


**Figure S12.** Double-layer capacitance (C_dl_) of catalysts synthesized under different calcination temperatures, determined from cyclic voltammetry (CV) measurements at various scan rates.


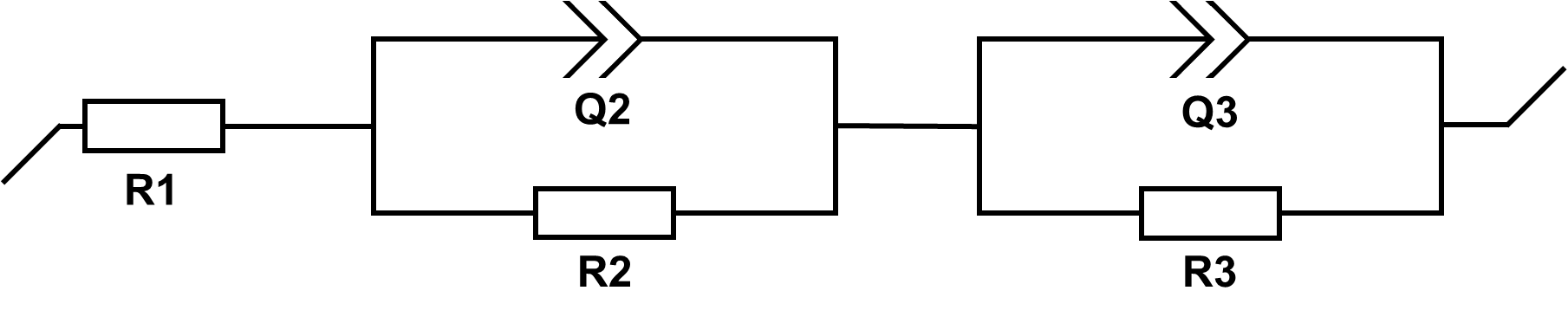


**Figure S13.** Equivalent circuit model used to fit the EIS data, where R1 represents the solution resistance, Q­2 corresponds to the double-layer capacitance, and Q3 represents the pseudocapacitance associated with the redox transition of Ir-OH species during the OER.


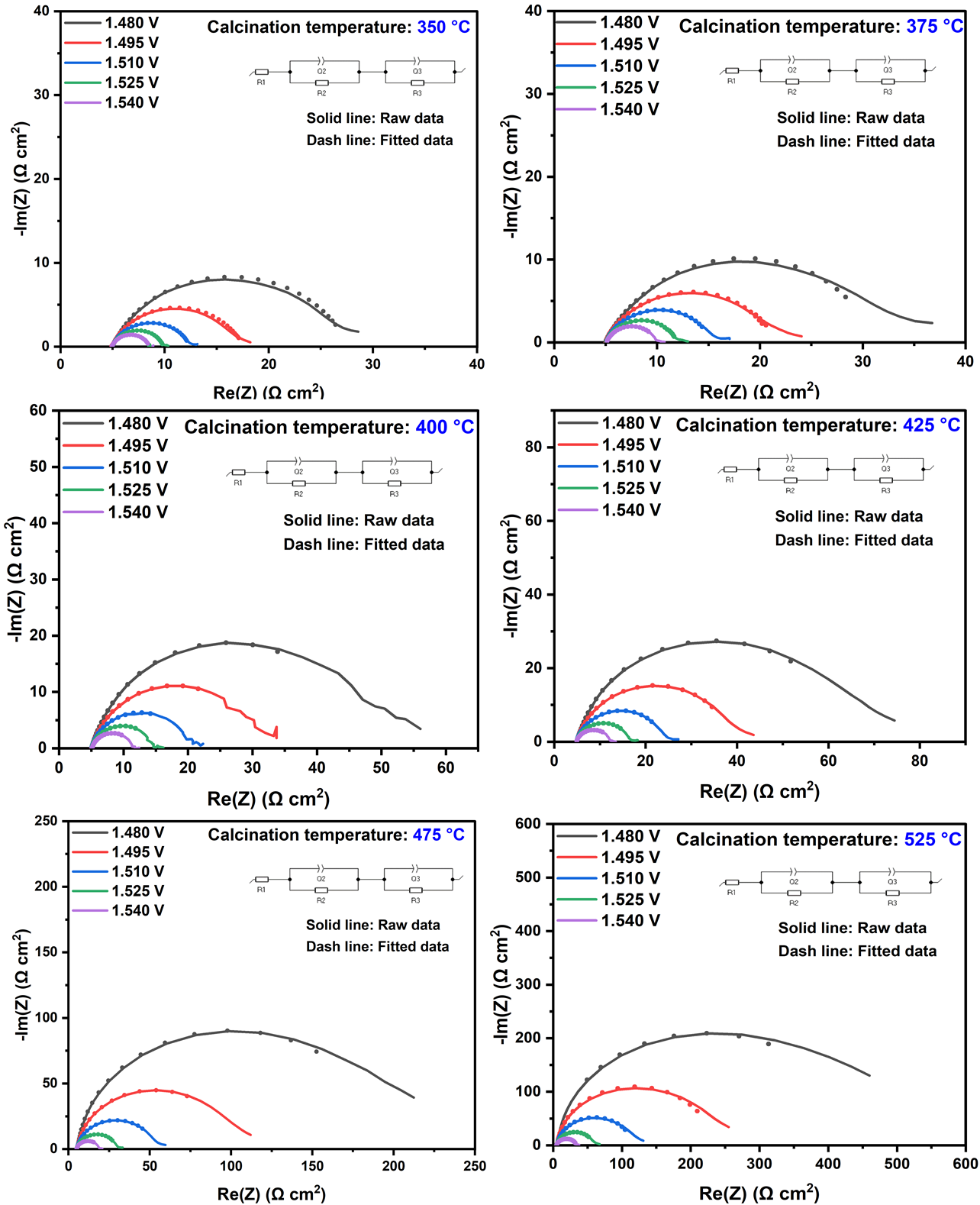


**Figure S14.** Potentiostatic electrochemical impedance spectroscopy (PEIS) raw spectra and fitted curves for catalysts prepared at different calcination temperatures.


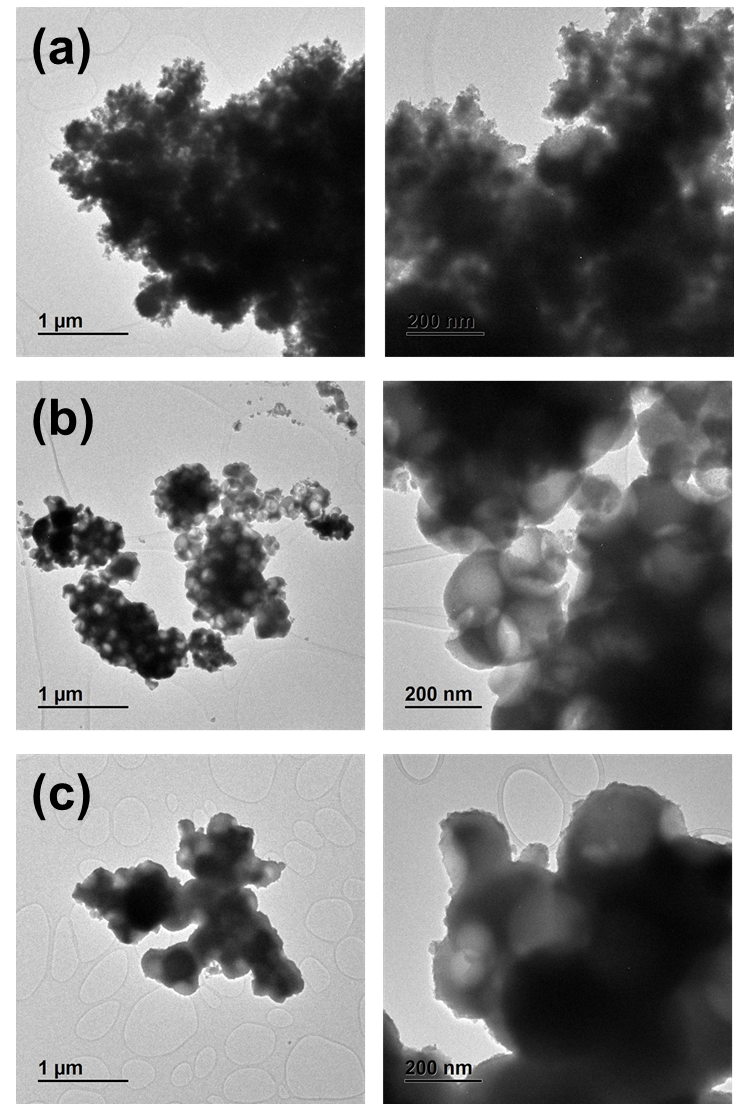


**Figure S15.** TEM analysis of M-IrO_x_ catalysts synthesized using PS beads of different diameters: (a) M-IrO_x_-190, (b) M-IrO_x_-240, and (c) M-IrO_x_-360.


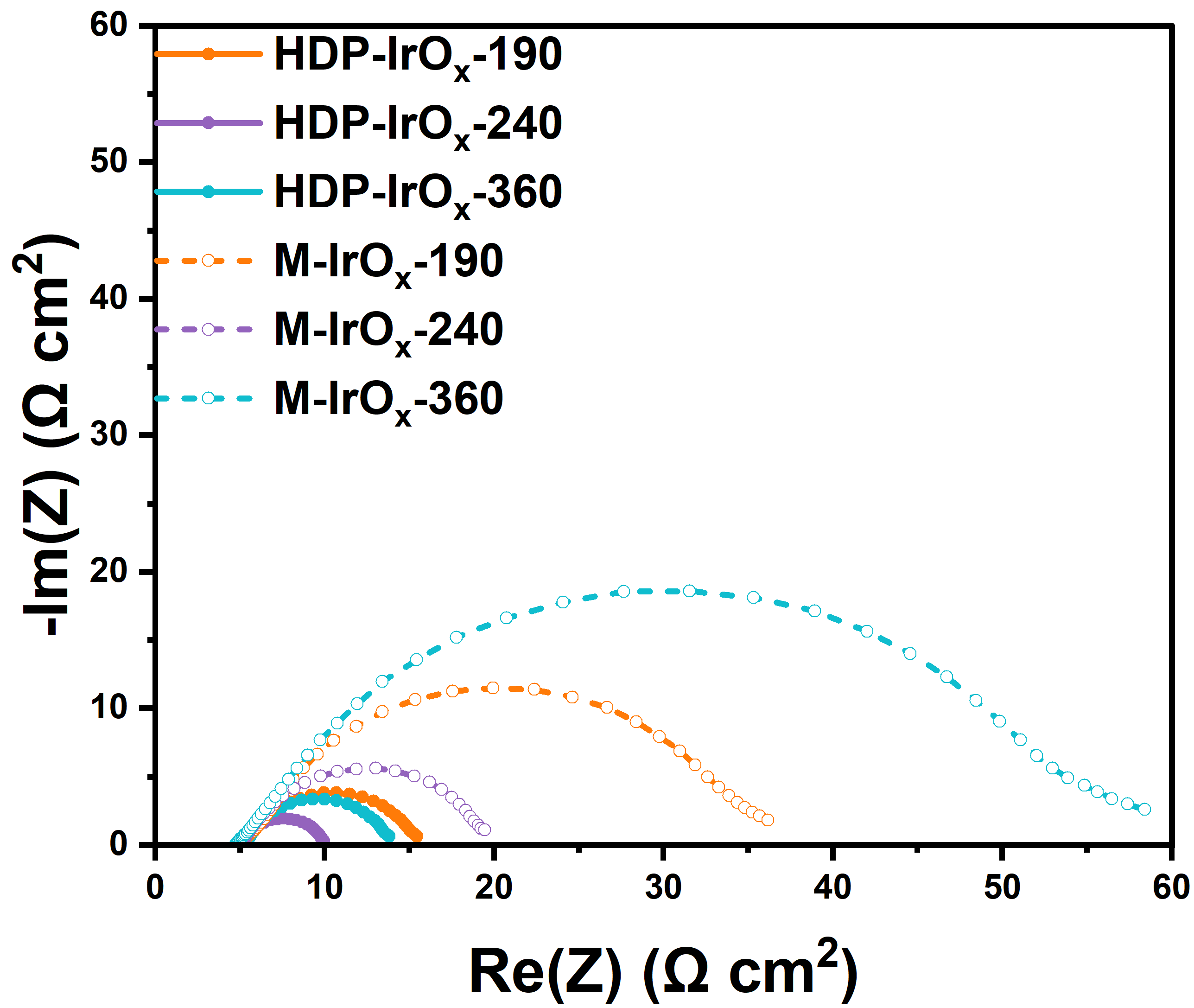


**Figure S16.** Nyquist plots obtained from PEIS measurements at 1.54 V for HDP IrOx and M-IrOx catalysts.


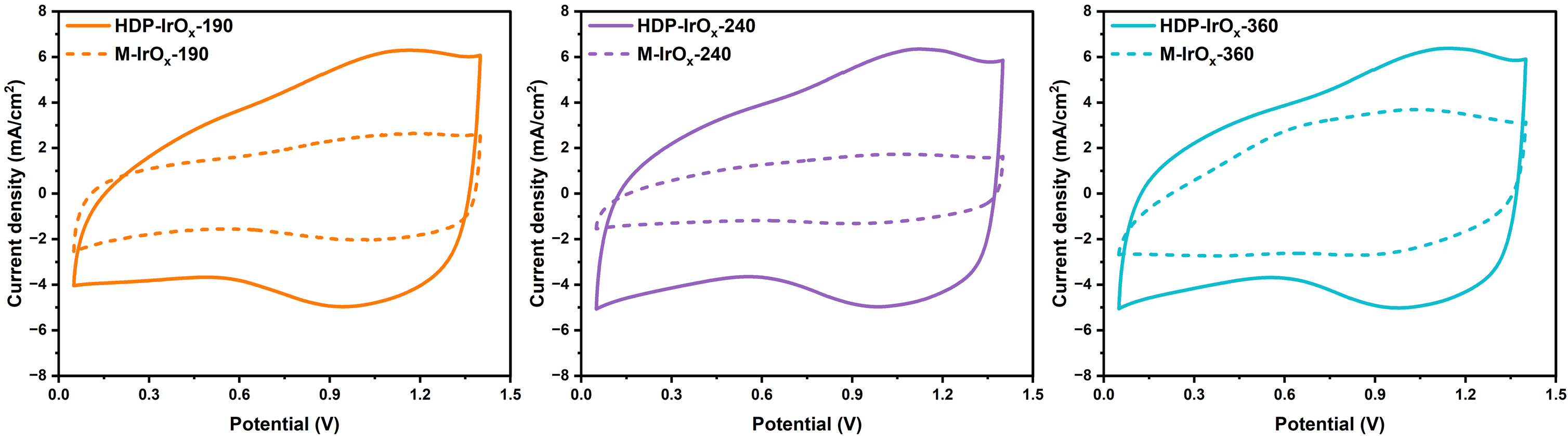


**Figure S17.** CV curves demonstrating the effect of dopamine coating.


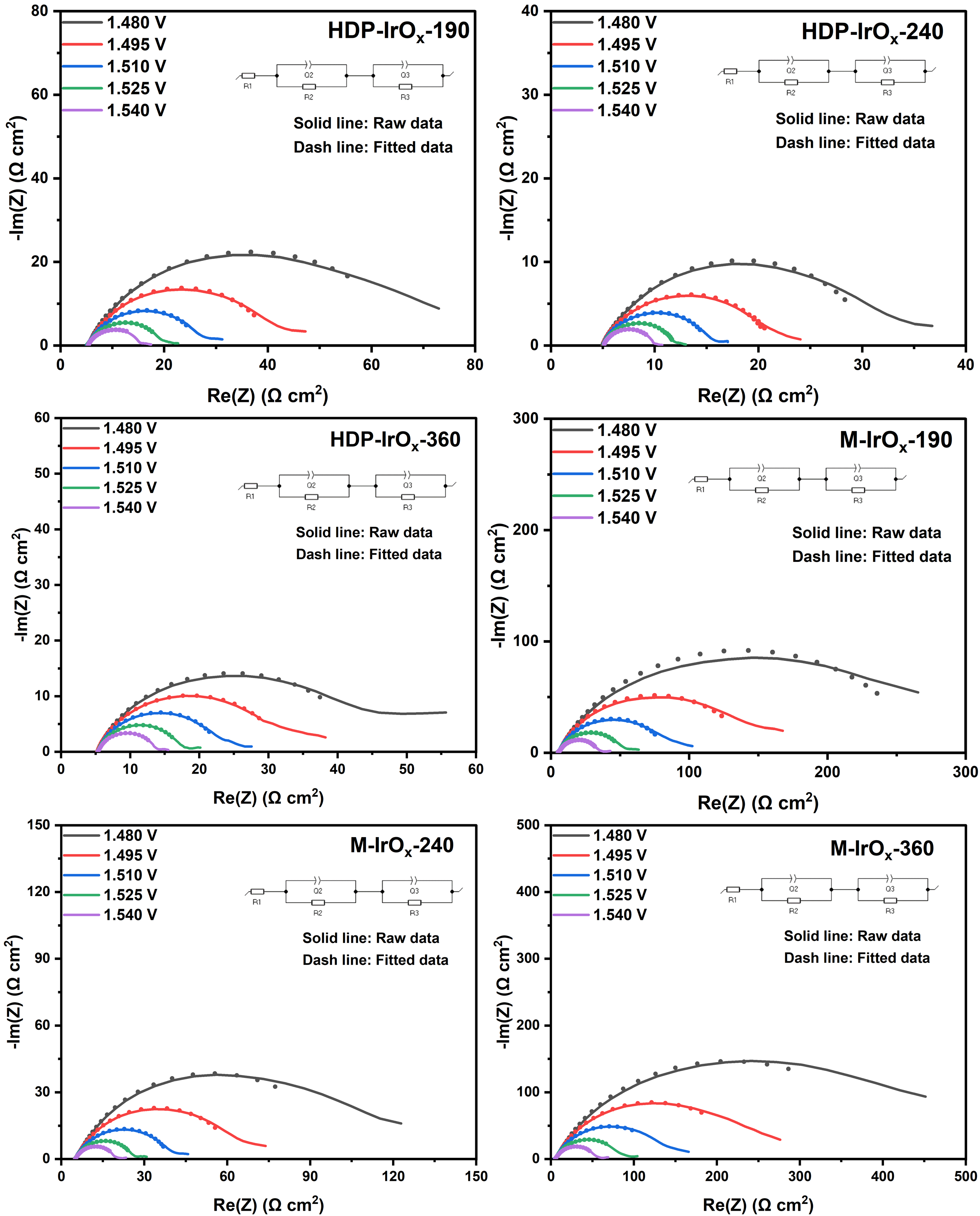


**Figure S18.** Potentiostatic electrochemical impedance spectroscopy (PEIS) raw spectra and fitted curves for each catalyst.


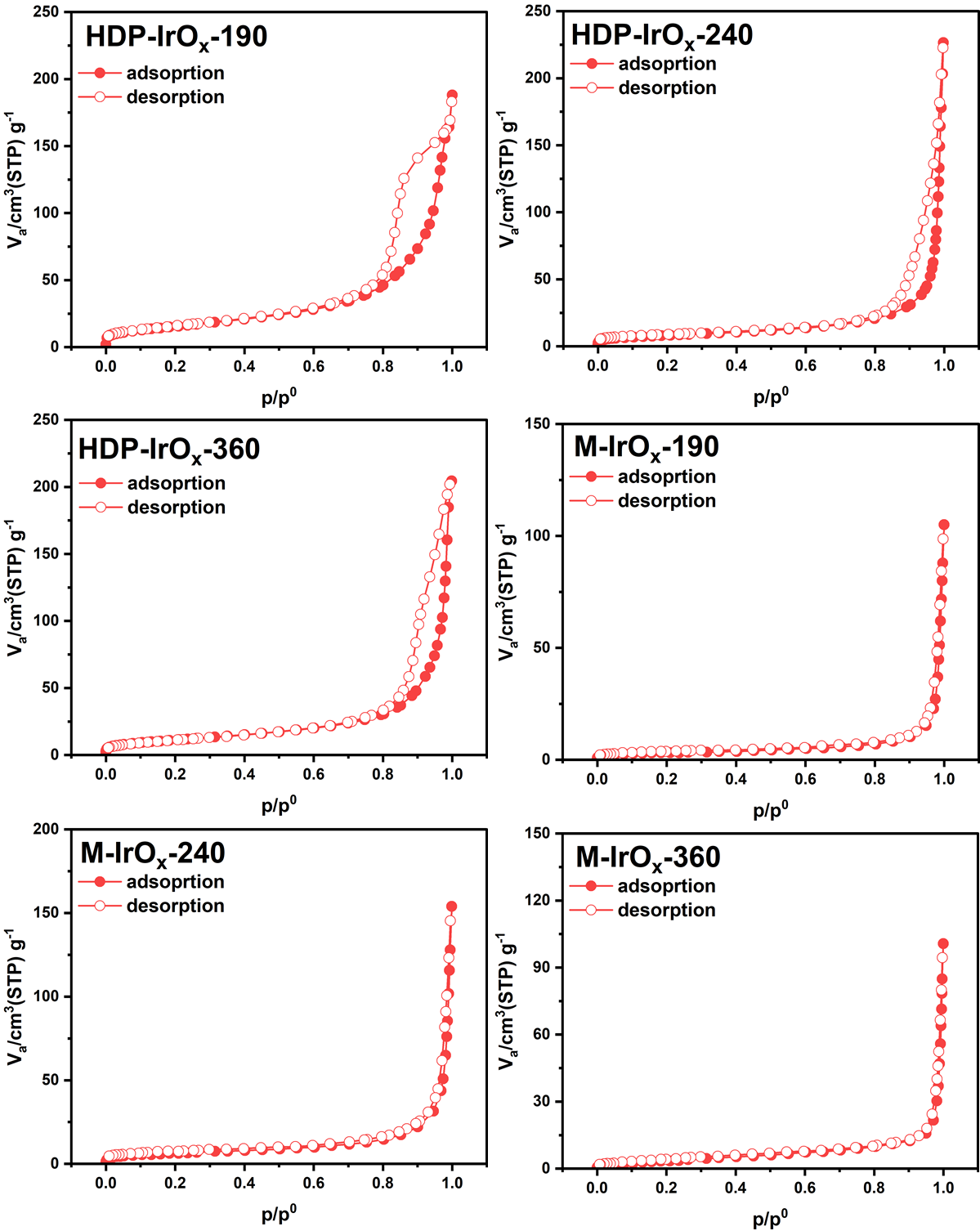


**Figure S19.** Nitrogen(N_2_) adsorption–desorption isotherms of each catalyst.


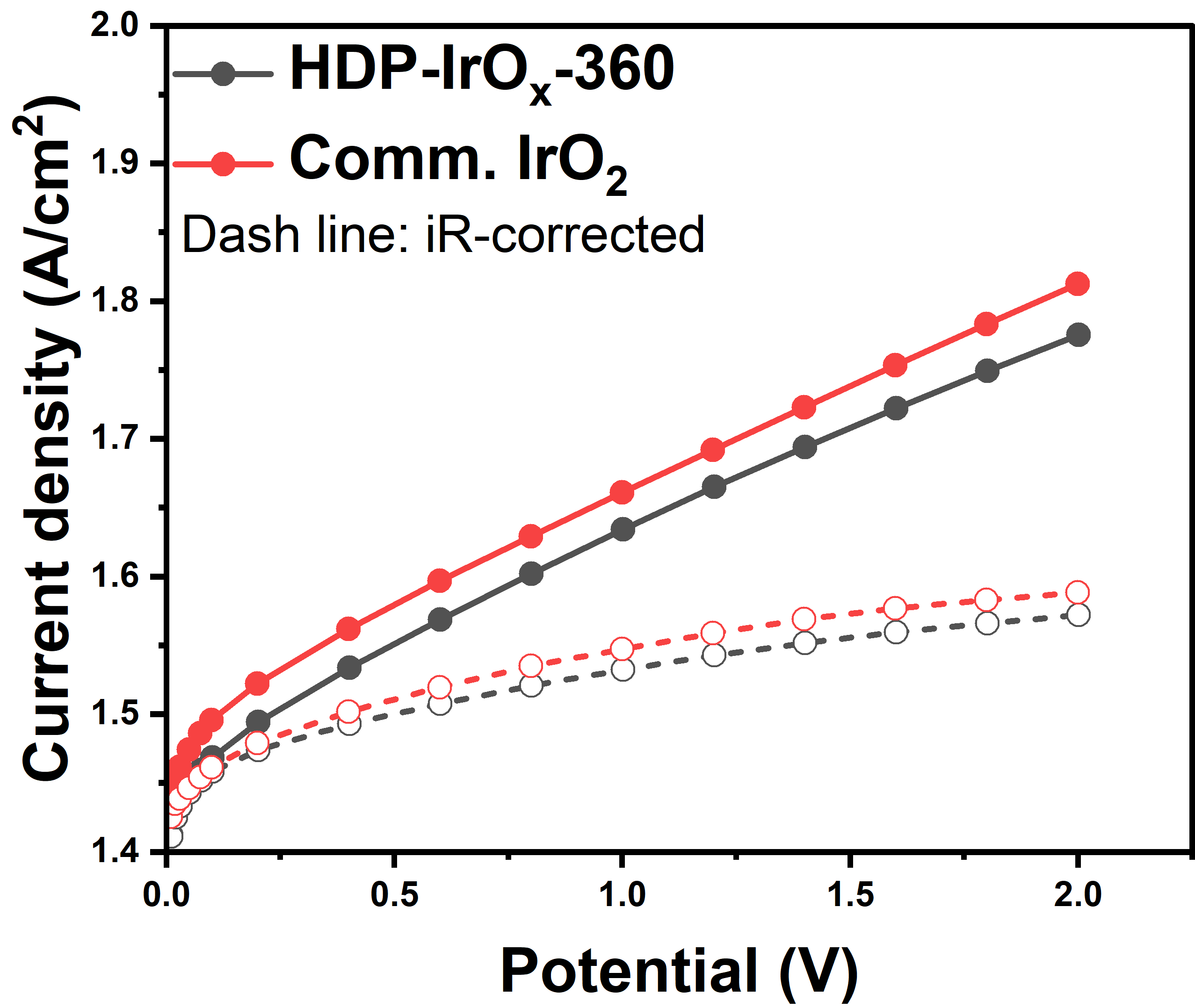


**Figure S20.** Polarization curves of HDP-IrO_x_-360 and commercial IrO_2_ measured in a single-cell configuration (solid lines: raw data; dashed lines: iR-corrected).


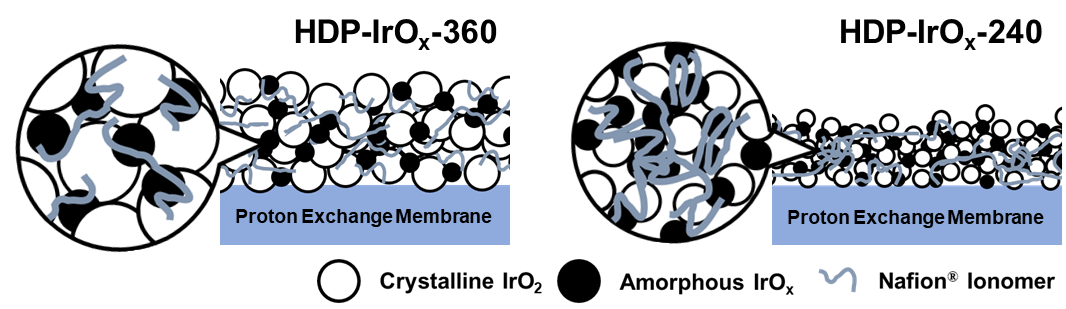


**Figure S21.** Template size-dependent distribution of catalyst and ionomer within the electrode.


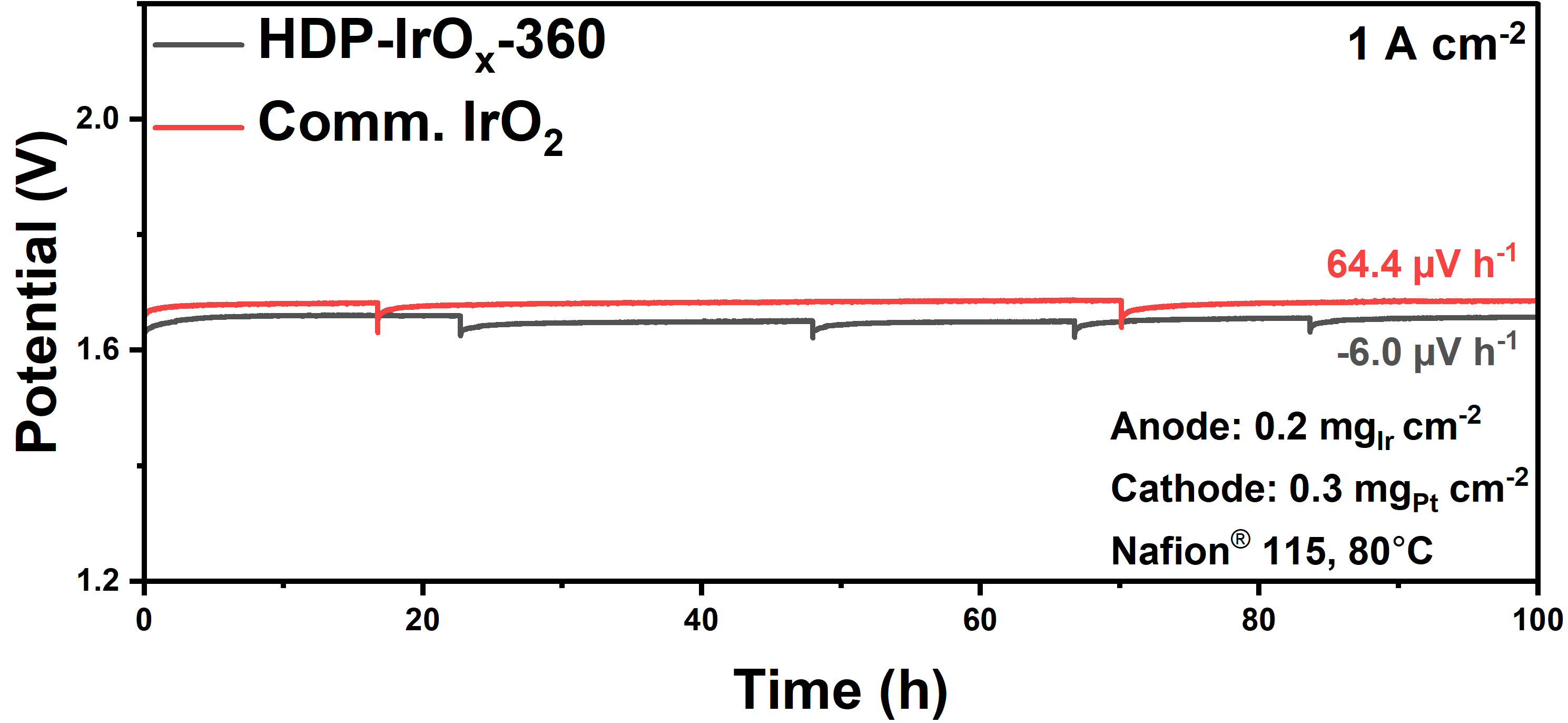


**Figure S21.** Chronopotentiometry durability test of HDP-IrOx-360 and commercial IrO_2_ in a single cell.


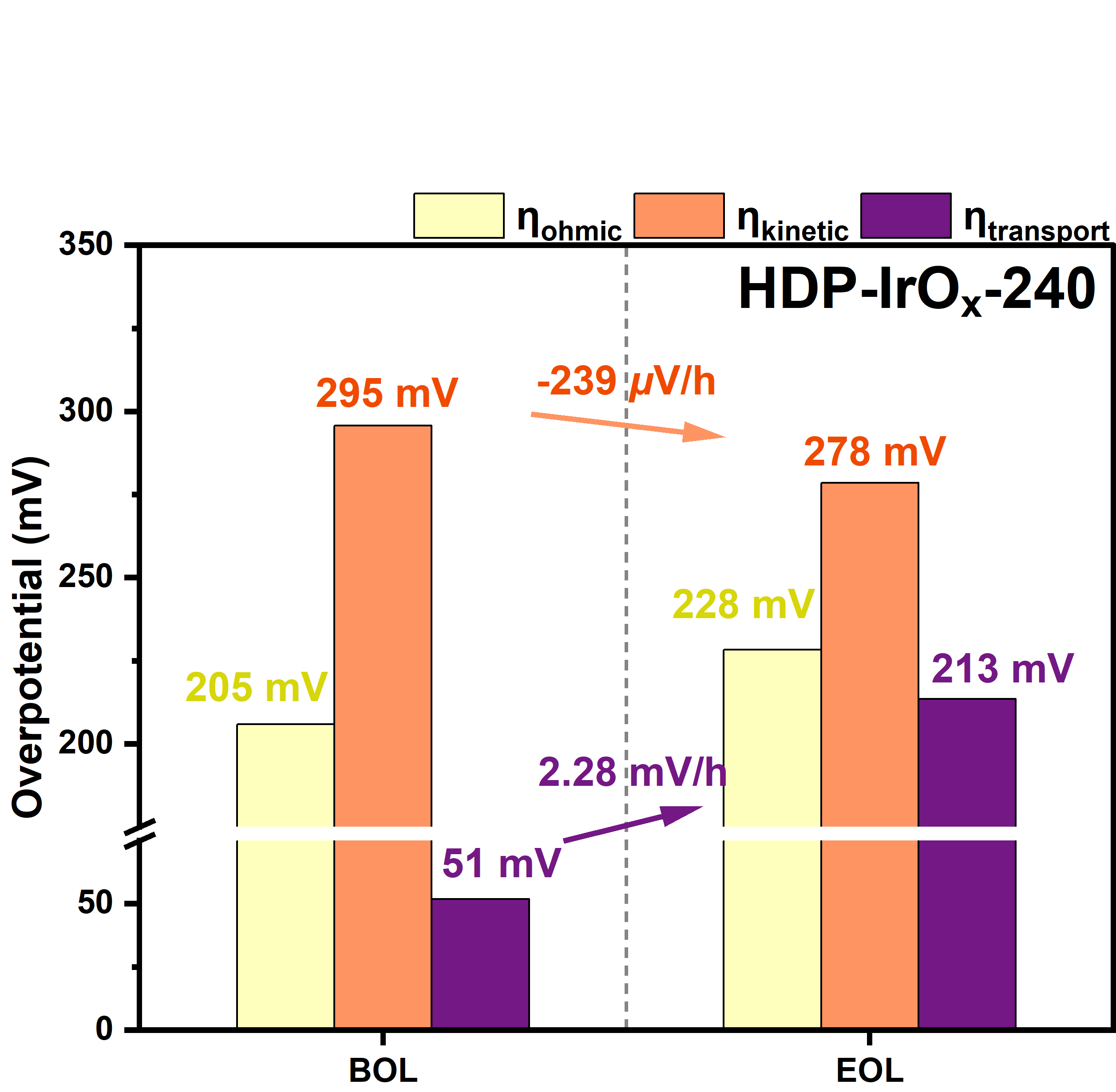


**Figure S23.** Comparison of overpotential components (ohmic, kinetic, and mass transport) BOL, and after EOL test for HDP-IrO_x_-240 at 2 A cm^-2^.


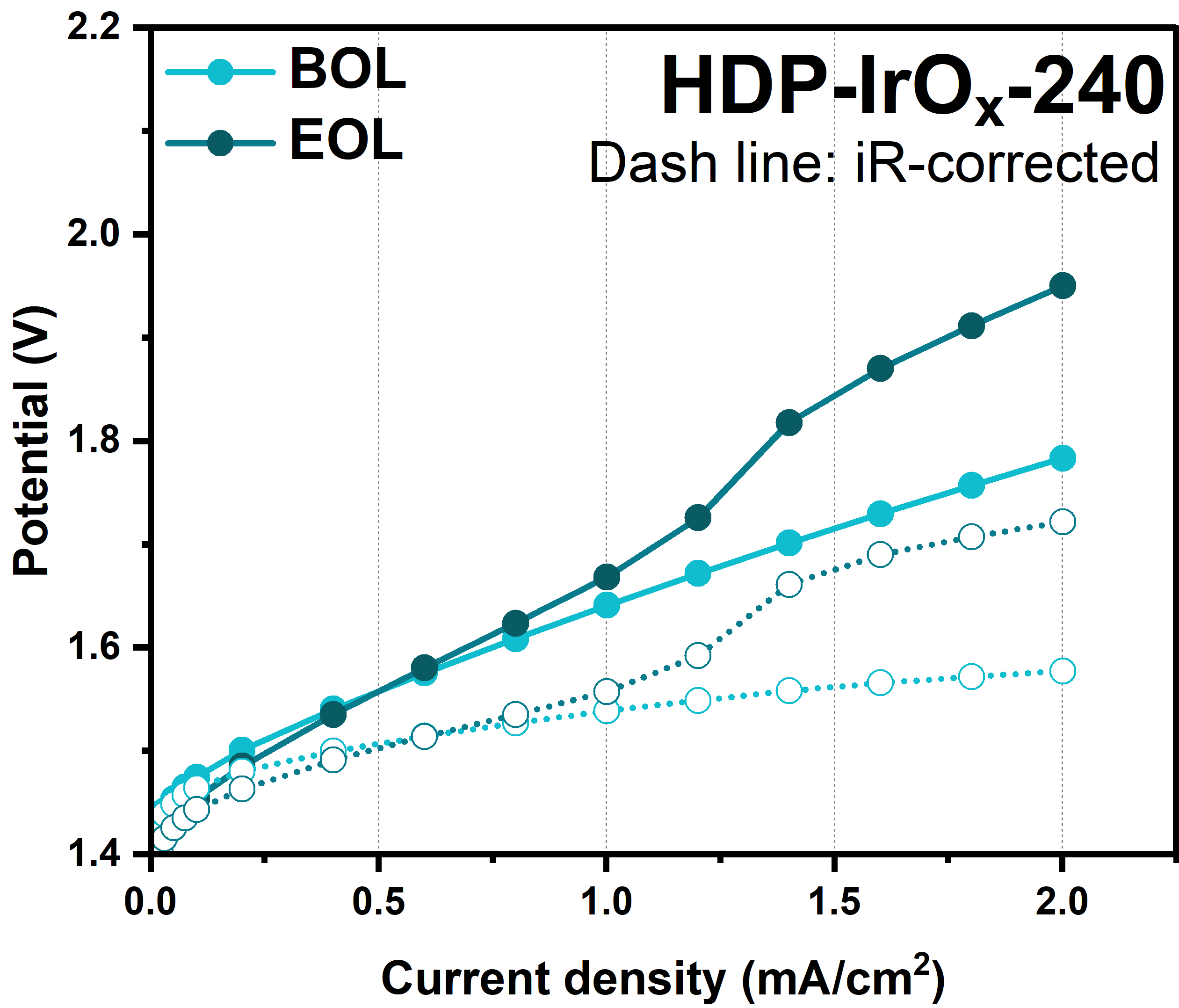


**Figure S24.** Comparison of iR-corrected polarization curves of HDP-IrO_x_-240 at BOL and EOL.

**Table S1**. Comparison of ECSA (derived from CV) and SSA of IrOx catalysts with different calcination temperatures.

| Calcination Temperature  [℃] | ECSA by CV  [m^2^ g^-1^] | SSA by BET equation  [m^2^ g^-1^] |
| --- | --- | --- |
| 350 | 2440.12 | 50.75 |
| 375 | 3198.38 | 36.46 |
| 400 | 1977.26 | 41.52 |
| 425 | 1734.44 | 39.46 |
| 475 | 913.74 | 47.00 |
| 525 | 599.54 | 32.91 |

**Table S2**. Comparison of degradation rates of Ir-based catalysts for PEM water electrolysis.

| Catalyst | PGM loading  [mg cm^-2^] | Anode loading [mg cm^-2^] | Cathode loading [mg cm^-2^] | Membrane | Temperature [℃] | Durability test | Degradation rate  [μV h^-1^] | Reference |
| --- | --- | --- | --- | --- | --- | --- | --- | --- |
| HDP-IrO_x_-360 | 0.5 | 0.2 | 0.3 | N115 | 80 | 1 A cm^-2^ @1036h | 31.5 | This study |
| HDP-IrO_x_-240 | 0.5 | 0.2 | 0.3 | N115 | 80 | 1 A cm^-2^ @77h | 274.1 | This study |
| IrO_2_ | 0.5 | 0.2 | 0.3 | N115 | 80 | 1 A cm^-2^ @100h | 64.4 | This study |
| IrO_2_ | 0.7 | 0.2 | 0.5 | N115 | 80 | 1 A cm^-2^ @57h | 8280 | [6] |
| IrO_2_-9La | 2.0 | 1.0 | 1.0 | N115 | 60 | 1 A cm^-2^ @200h | 1470 | [7] |
| IrO_x_-3Nd | 2.1 | 1.7 | 0.4 | N115 | 80 | 1 A cm^-2^ @1000h | 55.8 | [8] |
| IrO_2_ | 0.6 | 0.2 | 0.4 | N117 | 80 | 1 A cm^-2^ @100h | 2000 | [9] |
| IrO_x_·nH_2_O | 5.0 | 2.0 | 3.0 | N115 | 60 | 1 A cm^-2^ @600h | 5.9 | [10] |
| Ir-Sn PSC | 1.0 | - | - | N117 | 80 | 1 A cm^-2^ @96h | 882 | [11] |
| Ir/TiO_2_-MoO_x_ | 1.0 | 0.5 | 0.5 | N115 | 80 | 1 A cm^-2^ @50h | 2200 | [12] |
| Mn-RuIr | 1.8 | 1.0 ^a)^ | 0.8 | N115 | 80 | 0. 1 A cm^-2^ @10h | 4600 | [13] |
| IrO_x_@Ir/Ce-Nb_2_O_5_ | 0.6 | 0.3 | 0.3 | N115 | 65 | 1 A cm^-2^ @200h | 54.71 | [14] |
| ICM_activated_ | 0.68 | 0.28 | 0.4 | N212 | 80 | 1 A cm^-2^ @400h | 42 | [15] |
| Ru_0.8_Ir_0.2_Ox | 2.42 | 1.86 ^a)^ | 0.56 | N117 | 60 | 0.5 A cm^-2^ @100h | 89 | [16] |
| Ir/WO_3_@TiN | 0.8 | 0.3 | 0.5 | N115 | 80 | 1 A cm^-2^ @200h | 100 | [17] |
| (RuIr)O_2_/C | 1.23 | 0.25 ^a)^ | 1 | N212 | 80 | 1 A cm^-2^ @250h | 80 | [18] |
| Ir@WO_x_ NRs-100 | 0.54 | 0.14 | 0.4 | N115 | 80 | 1 A cm^-2^ @1030h | 49.7 | [19] |
| IrO_x_/Nb_4_N_5_-40 | 1.7 | 1.2 | 0.5 | N115 | 80 | 1 A cm^-2^ @150h | 600 | [20] |
| Ir@CeO_2_ | 0.5 | 0.3 | 0.2 | N115 | 80 | 1 A cm^-2^@1000h | 4.85 | [21] |

^a)^ Calculated as the sum of Ir and Ru loadings.

Reference

1. C. Roiron, C. Wang, I. V. Zenyuk, P. Atanassov, *J. Phys. Chem. Lett.* **2024**, 15, 11217.
2. Z. Zhou, W. Q. Zaman, W. Sun, L.-m. Cao, M. Tariq, J. Yang, *Chem. Commun.* **2018**, 54, 4959.
3. S. W. Lee, C. Baik, D.-H. Kim, C. Pak, *J. Power Sources* **2021**, 2021493, 229689.
4. Johnson Matthey. (2025). Pgm Market Report March 2025., https://www.platinum.matthey.com/documents/market-review, accessed.
5. S. Li, L. Deng, S. F. Hung, S. Zhao, L. Wang, Y. Hao, Y. Long, B. Li, Y. H. Hsu, Y. Y. Chen, Y. Zhang, T. Y. Chen, F. Hu, L. Li, Y. Hu, Y. Wu, S. Peng, *Adv. Mater.* **2025**, e07340.
6. J. Y. Choi, J. G. Kim, H. J. Lee, C. Pak, *Int. J. Hydrogen Energy* **2025**, 97, 57.
7. B. Zhou, X. Liu, L. Li, M, Liu, H. Liao, Y. Yu, F. Liu, P. Tan, J. Pan, *Chem. Eng. J.* **2025**, 521, 166886
8. N. Zhang, X. Liu, H. Zhong, W. Liu, D. Bao, J. Zeng, D. Wang, C. Ma, X. Zhang, *Angew. Chem.* **2025**, 137, e202503246
9. D. Wang, F. Lin, H. Luo, J. Zhou, W. Zhang, L. Li, Y. Wei, Q. Zhang, L. Gu, Y. Wang, M. Luo, F. Lv, S. Guo, *Nat. Commun.* **2025**, 16, 181
10. J. Xu, H. Jin, T. Lu, J. Li, Y. Liu, K. Davey, Y. Zheng, S. Z. Qiao, *Sci. Adv.*  **2023**, 9, 25
11. X. Zheng, J. Yang, P. Li, Q. Wang, J. Wu, E. Zhang, S. Chen, Z. Zhuang, W. Lai, S. Dou, W. Sun, D. Wang, Y. Li, *Sci. Adv.* **2023**, 9, 42
12. E. J. Kim, J. Shin, J. Bak, S. J. Lee, K. H. Kim, D. H. Song, J. H. Roh, Y. Lee, H. W. Kim, K. S. Lee, E. A. Cho, *Appl. Catal. B* **2021**, 280, 119433
13. J. Joo, Y. Park, J. Kim, T. Kwon, M. Jun, D. Ahn, H. Baik, J. H. Jang, J. Y. Kim, K. Lee *Small Methods* **2022**, 6, 2101236
14. Z. Gu, Z. Dou, Y. Chen, Y. Zhu, Y. Chen, G. Wang, Z. Zou, Q. Cheng, H. Yang, *J. Power Sources* **2026**, 661, 238652
15. W. Ko, J. Shim, H. Ahn, H. J. Kwon, K. Lee, Y. Jung, W. H. Antink, C. W. Lee, S. Heo, S. Lee, J. Jang, J. Kim, H. S. Lee, S. P. Cho, B. H. Lee, M. Kim, Y. E. Sung, T. Hyun, *J. Am. Chem. Soc.* **2025**, 147, 2369
16. Y. Yuan, H. Fang, K. Chen, J. Huang, J. Chen, Z. Lu, H. Wang, Z. Zhao, W. Chen, Z. Wen, *Adv. Mater.* **2025**, 37, 2501607
17. P. Fang, Y. Wang, F. Zhang, Z. Zhang, R. Qin, Y. Su, L. Kong, J. Gao, Y. Chen, Y. Li, *Adv. Funct. Mater.* **2025**, 35, 2501142
18. Y. Park, H. Y. Jang, T. K. Lee, D. Kim, D. Kim, H. Baik, J. Choi, T. Kwon, S. J . Yoo, S. Back, K. Lee, *Nat. Commun* **2025**, 16, 579
19. G. Jiang, H. Yu, Y. Li, D. Yao, J. Chi, S. Sun, Z. Shao, *ACS Appl. Mater. Interfaces* **2021**, 13, 15073
20. X. Duan, H. Liu, W. Zhang, Q. Ma, Q. Xu, L. Khotseng, H. Su, *Electrochim. Acta* **2023**, 470, 143271
21. X. Mao, M. Zhu, M. Xie, G. Zou, Y. Kuang, S. Guo, J. Hu, X. Xu, *Nano Lett.* **2025**, 25, 16253
